# Efficacy of resistance training in hypoxia on muscle hypertrophy and strength development: a systematic review with meta-analysis

**DOI:** 10.1038/s41598-023-30808-4

**Published:** 2023-03-04

**Authors:** Cristina Benavente, Brad J. Schoenfeld, Paulino Padial, Belén Feriche

**Affiliations:** 1 Department of Physical Education and Sport, Faculty of Sport Sciences, University of Granada, Granada, Spain; 2 Department of Exercise Science and Recreation, CUNY Lehman College, The Bronx, NY USA

**Keywords:** Biophysics, Physiology

## Abstract

A systematic review and meta-analysis was conducted to determine the effects of resistance training under hypoxic conditions (RTH) on muscle hypertrophy and strength development. Searches of PubMed-Medline, Web of Science, Sport Discus and the Cochrane Library were conducted comparing the effect of RTH versus normoxia (RTN) on muscle hypertrophy (cross sectional area (CSA), lean mass and muscle thickness) and strength development [1-repetition maximum (1RM)]. An overall meta-analysis and subanalyses of training load (low, moderate or high), inter-set rest interval (short, moderate or long) and severity of hypoxia (moderate or high) were conducted to explore the effects on RTH outcomes. Seventeen studies met inclusion criteria. The overall analyses showed similar improvements in CSA (SMD [CIs] = 0.17 [− 0.07; 0.42]) and 1RM (SMD = 0.13 [0.0; 0.27]) between RTH and RTN. Subanalyses indicated a small effect on CSA for shorter inter-set rest intervals, moderate hypoxia and moderate loads favoring RTH. Moreover, a medium effect for longer inter-set rest intervals and a trivial to small effect for severe hypoxia and moderate loads favoring RTH was found on 1RM. Evidence suggests that RTH employed with moderate loads (60–80% 1RM) enhances both hypertrophy and strength. Hypertrophy appears to benefit from shorter (≤ 60 s) inter-set rest intervals during RTH while greater gains in strength are achieved with longer rest intervals (≥ 120 s). The use of moderate hypoxia (14.3–16% FiO_2_) seems to be somewhat beneficial to hypertrophy but not strength. Further research is required with greater standardization of protocols to draw stronger conclusions on the topic.

## Introduction

Optimizing training methods to efficiently enhance muscle hypertrophy and strength is of primary interest to athletes and health/fitness practitioners^1,2^. During the last decade, the combination resistance training (R_T_) under hypoxic conditions has become an area of great research interest due to its potentially beneficial effects on muscular adaptations^3–8^. Moderate to severe intermittent hypoxic resistance training (RTH) performed for 4 to 8 weeks at simulated hypoxia is currently the most studied strategy related to this topic^9,10^.

Based on the effects of hypoxia-induced increases in metabolic stress, anabolic hormones, cytokines, and/or cellular swelling, among others^4,6,9,11,12^, RTH conceivably could promote greater improvements in muscle size and strength than the same training regimen carried out in normoxia (RTN)^11,13,14^. Conceivably, these improvements could therefore be achieved under hypoxic conditions in a shorter time period compared to training under normoxic conditions^5,15^. Nevertheless, discrepancies in the available studies among training protocols (from 3 to 6 sets; low, moderate or heavy loads; from 30 to 180 s of inter-set rest), training period lengths (from 3 to 8 weeks), severity of hypoxia (from 12 to 16% of the inspired fraction of oxygen (FiO_2_), participant training levels (untrained, recreational trained, recreational resistance trained, strength trained, well-trained in a sport discipline and professional athletes) or session type and muscles worked (isolated small or big muscles vs full-body sessions), make it difficult to draw firm conclusions on the potential benefit of RTH versus RTN^10,16,17^.

Two recently published narrative reviews^10,17^ and one meta-analysis^16^ that analysed the effect of RTH on muscle hypertrophy and strength development shed light on this topic. In particular, the meta-analysis by Ramos-Campo et al.^16^ pooled nine studies that compared the effects of RTH versus RTN on muscle cross-sectional area, lean mass and strength. The standardised mean differences (SMD) reported between RHT and RTN did not clearly favor the hypoxia conditions (SMD = 0.24 [− 0.19; 0.68] and 0.20 [− 0.13; 0.53] for CSA and 1RM, respectively). Moreover, the authors established different subgroups for upper and lower limbs as a possible heterogeneity bias, but results indicated similar training effects in CSA and 1RM between environmental conditions (RTH vs. RTN) for this variable. Unfortunately, previously discussed methodological discrepancies between studies were not assessed due to the reduced number of available data up to that date, which potentially limited the ability to draw practical inferences. Moreover, since 2017, several studies on the topic have been published, thereby providing an opportunity to achieve greater statistical power when meta-analyzing data on the effects of hypoxia in R_T_. The recent narrative review by Deldicque^17^ includes 16 studies and extends the discussion of RTH to performance-based outcomes such as velocity and muscular power. Given its narrative format, however, this review did not seek to quantify the effect of RTH on muscular adaptations and its non-systematic approach introduces the potential for selection bias^18^, thereby limiting the veracity of its conclusions.

The aim of this paper was to perform an updated systematic review and meta-analysis to explore the effect of RTH on muscle hypertrophy and strength development. To address the gaps in the current literature, we also subanalyzed the potential impact of methodological covariates such as the training load, inter-set rest interval, and the severity of hypoxia. The analysis thus enhances our ability to provide specific recommendations about the effectiveness of strength training in hypoxia, as well as to detect procedural gaps in the literature that hopefully spurs future research on the topic.

## Methods

### Study design

This meta-analysis followed the recommendations described in the Preferred Reporting Items for Systematic Reviews and Meta-Analyses (PRISMA) guidelines^19^ (see Tables S1 and S2).

### Data sources and search strategy

A systematic search of relevant studies was performed using PubMed-Medline, Web of Science, Sport Discus and the Cochrane Library from database inception through 7 June 2022. The following combination of terms was used for the search: (“strength training” OR “resistance training” OR “weight training”) AND (“hypoxia” OR “altitude” OR “hypoxic training”) (see Table S3 for specific search strategies), without a restriction of date of publication. The search was performed individually by two authors (CB and BF). Full texts of studies deemed potentially relevant based on title and abstract were screened, and a final decision was then made as to whether a study warranted inclusion. Any discrepancies were resolved through discussion; if needed, a third author (PP) arbitrated to arrive at a final decision.

### Inclusion criteria

We included studies that (1) examined the effect of R_T_ under intermittent terrestrial or simulated hypoxia for at least 3 weeks^20^ on muscle hypertrophy (cross-sectional area [CSA], lean mass or muscle thickness) and strength development (via one maximum repetition, 1RM) in healthy individuals between 18 and 65 years of age using a randomized design; (2) included a normoxic control group; (3) were published in English-language peer-reviewed journals; and (4) provided information about outcomes both at baseline and post-study. Research studies were excluded if they (1) were not original investigations published in full; (2) did not specify the tests to be evaluated; (3) applied hypoxia via local techniques, such as blood flow restriction; (4) did not provide or specify numerical or graphic data; and (5) examined only the acute effects of interventions.

### Data extraction and study outcomes

Data extraction of the included studies was conducted in a standardised manner by two authors independently (CB and BF). To ensure the reliability of this process, each author performed the data extraction of all studies included in the meta-analyses and separately entered the data into a spreadsheet. The data were then crosschecked and combined into a single spreadsheet for analysis.

For each included study we extracted the following data: authors and year of publication; sample size and mean age and weight of participants for each group; the type of hypoxic environment; the FiO_2_; and the training status (untrained: subjects were not involved in regular resistance training program for at least 6 months before the study^21^; trained: participants achieved at least 12 months continuous resistance training history immediately prior to the study^15^; in the absence of a specific description of participant training status, we defaulted to the description provided by the authors). The information extracted about the characteristics of the R_T_ programs included: training frequency (sessions/week), relative load lifted, sets, proximity to failure, inter-set rest interval, the type of exercise and the outcomes measured (i.e., maximal strength, CSA and/or lean mass and/or muscle thickness) (Table 1). In cases where data were not sufficiently reported, we contacted the authors of the relevant studies for additional information. In cases where an article presented results using figures, two authors (CB and BF) extracted the values of the outcomes using online software (WebPlotDigitizer)^22^. When disagreement reached 3%, a third investigator (PP) extracted the data with the online software. The mean of the two closest derived assessments was used for analysis.

**Table 1:** Main characteristics of included studies in the meta-analysis.

Study	n	H condition	Effective FiO_2_	Training level	Age (years)	Weight (Kg)	Training intervention			Variables measured	
							Weeks (s/w)	Methodology	Exercise	Strength development	Muscle hypertrophy
Chycki et al.^40^	6 (M)	NH (chamber)	12.9% FiO_2_	Rec. resistance trained	21 ± 2.4	80.6 ± 12.3	6 (2)	8 sets × 10 reps. 70%RM (Rest 180 s)	Bench press Barbell Squat		Lean mass
	6 (M)	N (chamber)	21% FiO_2_		22 ± 1.5	81.1 ± 7.5					
Fashi and Ahmadizad^47^	7 (M)	NH (gas)	12,7% FiO_2_	Untrained	21 ± 4		4 (3)	3 sets x reps. to failure. 50% 10RM (~37% 1RM) (Rest 60 s)	Back squat	RM	CSA
	7 (M)	N (gas)	20,9% FiO_2_								
Friedman et al.^3^	10 (M)	NH (room)	12% FiO_2_	Untrained and recreational	25.1 ± 2.9	77.0 ± 9.0	4 (3)	6 sets × 25 reps. 30%RM (Rest 60 s)	Knee extension		CSA
	9 (M)	N (room)	21% FiO_2_		24.3 ± 2.5	72.9 ± 9.0					
Ho et al.^21^	9 (M)	NH (chamber)	15% FiO_2_	Rec. trained	21.4 ± 2.2	66.5 ± 8.2	6 (3)	3 sets × 10 RM (75% RM) (Rest 120 s)	Squat	RM	Lean mass
	9 (M)	N (chamber)	21% FiO_2_		21.2 ± 1.9	67.9 ± 9.5					
Honda et al.^41^	9 (M)	NH (room)	14.4% FiO_2_	Untrained and recreational	29 ± 5	68.2 ± 6.7	8 (2)	5 sets × 10 reps. 70%RM (Rest 90 s)	Bench press Leg press	RM	Lean mass
	7 (M)	N (room)	21% FiO_2_		29 ± 4	65.8 ± 9.7					
Inness et al.^15^	10 (M)	NH (facemask)	14.3% FiO_2_	Strength trained	Bt 18–34	83.1 ± 7.5	7 (3)	2–4 sets × 3–6 reps. 75%RM (Rest 180 s)	Squat Deadlift Lunge	RM	
	10 (M)	N (facemask)	20% FiO_2_			80.2 ± 12.0					
Kon et al.^46^	9 (M)	NH (room)	14.4% FiO_2_	Rec. resistance trained	28.4 ± 1.6	68.2 ± 2.2	8 (2)	5 sets × 10 reps. 70% RM (Rest 90 s)	Bench press Leg press	RM	CSA Lean mass
	7 (M)	N (room)	21% FiO_2_		28.2 ± 1.4	65.8 ± 3.7					
Kurobe et al.^6^	6 (M)	NH (room)	12.7% FiO_2_	Untrained	23.0 ± 1.0	60.2 ± 1.6	8 (3)	3 sets x reps. to failure. 10RM (75% RM) (Rest 60 s)	Elbow extensions	RM	Muscle thickness
	7 (M)	N (room)	20.9% FiO_2_								
Manimmanakorn et al.^37^	10 (F)	NH (facemask)	80% SpO_2_	Well-trained netball players	20.2 ± 3.3	65.2 ± 6.5	5 (3)	3 sets x reps. to failure. 20%RM (Rest 30 s bt. set and 120 s bt. exercises)	Knee flexion Knee extension		CSA
	10 (F)	N (facemask)	21% FiO_2_								
Martínez-Guardado et al.^45^	15 (M)	NH (chamber)	15% FiO_2_	Strength trained	24.6 ± 6.8	74.9 ± 11.5	8 (2)	3 rounds × 2 blocks x 3 × 6RM (85%RM) (Rest 35 s bt exercises, 180 s bt sets, 5 min bt blocks)	Bench press Leg extension Front pull down Deadlift Preacher curl Calf raises	RM	Lean mass
	13 (M)	N (chamber)	20.9% FiO_2_		23.2 ± 5.2	69.4 ± 7.4					
Martínez-Guardado et al.^39^	16 (M)	NH (chamber)	13% FiO_2_	Untrained	25.7 ± 6.4	74.7 ± 12.9	7 (3)	3 sets x reps. to failure. 65–75–80%RM (Rest 90 s)	Bench press Bicep’s curl French press Pendlay row Half squat	RM	Lean mass
	16 (M)	N (chamber)	21% FiO_2_			81.1 ± 11.7					
Mayo et al.^43^	8 (M)	NH (chamber)	14.4% FiO_2_	Professional rugby athletes	24 ± 3	98.7 ± 12.8	3 (4)	1–12 sets × 2–4 reps. 85–92.5%RM (Rest 180 s bt. super sets)	Back squat Bench press Weighted Chin-up	RM	
	9 (M)	N (chamber)	20.9% FiO_2_								
Nishimura et al.^5^	7 (M)	NH (room)	16% FiO_2_	Untrained	22.7 ± 2.7	66.8 ± 6.0	6 (2)	4 sets × 10 reps. 70% RM (Rest 60 s)	Elbow flexion Elbow extension	RM	CSA flexors CSA extensors
	7 (M)	N (outside)	21% FiO_2_		21.6 ± 1.6	65.0 ± 8.1					
Ramos-Campo et al.^38^	15 (M)	NH (chamber)	15% FiO_2_	Strength trained	24.6 ± 6.8	74.9 ± 11.5	8 (2)	3 rounds × 2 blocks × 3 sets x 6RM (85%RM) (Rest 35 s bt. exercises, 180 s bt. sets, 5 min bt. blocks)	Bench press Leg extension Front pull down Deadlift Preacher curl Calf raises		Muscle thickness
	13 (M)	N (chamber)	20.9% FiO_2_		23.2 ± 5.2	69.4 ± 7.4					
Törpel et al.^7^	20 (3F, 17M)	NH (facemask)	80–85% SpO_2_	Untrained	24.5 ± 4.5	75.5 ± 7.8	5 (4)	3 sets × 15 reps. 25–40% RM (Rest 30 s)	2 training plans with 8 machine-based resistance exercises		Lean mass
	17 (2F, 15M)	N (facemask)	20.9% FiO_2_		24.0 ± 3.6	76.3 ± 9.2					
van Doorslaer de ten Ryen et al.^42^	10 (M)	NH (chamber)	13.5% FiO_2_	Untrained	21.2 ± 0.5	73.3 ± 3.0	4 (3)	6 sets × 10 reps. 80% RM (Rest 120 s)	One leg extension	RM	Muscle thickness
	9 (M)	N (chamber)	21% FiO_2_		20.7 ± 0.8	71.1 ± 3.1					
Yan et al.^44^	8 (M)	NH (room)	12.6% FiO_2_	Rec. trained	22.2 ± 2.6	70.5 ± 10.0	5 (2)	5 sets × 10 reps. 70% RM (Rest 60 s)	Back squat	RM	Lean mass
	9 (M)	NH (room)	16% FiO_2_								
	8 (M)	N (room)	21% FiO_2_								

n: sample size; H: hypoxia; N: normoxia; NH: normobaric hypoxia; FiO_2_: fraction of inspired oxygen; SpO_2_: arterial oxygen saturation; s/w: sessions per week; reps.: repetitions; Bt: between; Rec.: recreationally; RM: repetition maximum; CSA: cross-sectional area; M: male; F: female.

To assess potential confounding from covariates, we carried out subanalyses of data on the effects of training load (low < 50% 1RM); moderate = 60–80% 1RM; heavy > 80% 1RM), inter-set rest interval (short < 60 s; moderate = 60 to < 120 s; long ≥ 120 s) and severity of hypoxia (moderate = 14.3 to 16% FiO_2_; severe < 14.3 to 11% FiO_2_).

### Evaluation of the methodology of the studies selected

All trials included in the meta-analysis were assessed for methodological quality using relevant items from the Cochrane's risk of bias tool^23^ and the PEDro scale^24^ by two authors (CB and BF) with a third investigator (PP) in case of disagreement. The assessment of the selected studies included specification of eligibility criteria, random sequence generation, allocation concealment, inter-group similarity in main study outcomes at baseline, blinding of participants, blinding of outcome, incomplete outcome data and selective reporting. Details of this quality assessment can be found in Table S4.

### Statistical analysis

All studies were independently analysed for each main outcome (CSA, lean mass and maximal dynamic strength [1RM]) using pre- and post-study results under hypoxic or normoxic conditions with values expressed as standardised mean differences (SMD) and their 90% confidence intervals (CIs). Subgroup analyses were performed to determine possible confounding effects of load, inter-set rest interval, and severity of the hypoxia. Standardised effect size coefficients from RCTs were computed as the mean differences between the mean change in hypoxic and normoxic groups from baseline to post-intervention, divided by the mean pooled baseline standard deviation^25^: $d=c(dfH,N)\cdot [((\overline{X }pre,H-\overline{X }pos,H)-(\overline{X }pre,N-\overline{X }pos,N))/(\overline{S }pre)]$. In the intra-group pre-post measurement, the mean change from baseline to post-intervention, divided by the pooled baseline standard deviation, was used to calculate the standardised effect size coefficient for each intervention group: $d=c(df)\cdot [(\overline{X }pre,H- \overline{X }pos,H)/Spre]$. Both coefficients included correction factors for small samples $c(dfH,N)$ and $c(df)$^26^. The inverse variance method was used in both cases for the weighting of studies (see Table S5). Additionally, we calculated the raw (unstandardised) mean difference for pre-post studies $(\overline{X }pre,H-\overline{X }pos,H)$ and RCTs $(\overline{X }pre,H-\overline{X }pos,H)-(\overline{X }pre,N-\overline{X }pos,N)$ by using the weights from our standardised meta-analysis to estimate the pooled mean difference in each outcome.

We analysed data using a multi-level random effects model to account for multiple effects nested within groups, studies and participants (three-level analysis)^27^. This approach allows to control for the bias of combining several measures from the same study (See r code as supplementary Fig. S1). Independent effect size coefficients from studies and outcomes were combined and analysed using the DerSimonian and Laird's^28^ random effects model. The weighted standardised mean change from baseline to post-intervention was the pooled effect size of each outcome. Consistent with previous meta-analytic approaches^29^, we chose to avoid drawing binary conclusions via traditional null hypothesis significance testing given the documented issues with this statistical method^30,31^. Rather, we considered the spectrum of possible estimates from the lower to upper limits of compatibility, placing the greatest inferential emphasis on the point estimate. Threshold values for SMDs were interpreted as: “trivial” (≤ 0.20); “small” (0.21–0.50); “medium” (0.51–0.80); and “large” (> 0.80)^32^.

For SMDs with a positive value, the reported result favors the RTH; conversely, results with a negative value favor RTN. The *Q* test and *I*^2^ index were calculated to estimate potential statistical heterogeneity. A threshold from 30 to 60% represented a moderate level of heterogeneity, *p* < 0.10. Potential small study bias was analysed using Egger's test^33^ as estimated from a funnel plot (Fig. S2). A sensitivity analysis was performed to control for the robustness in the outcomes that included the studies eliminated for undetermined inter-set rest periods^34^. All statistical analyses were performed using the Metafor package^35^ in the R statistics program^36^.

## Results

### Study selection

**Figure 1:**
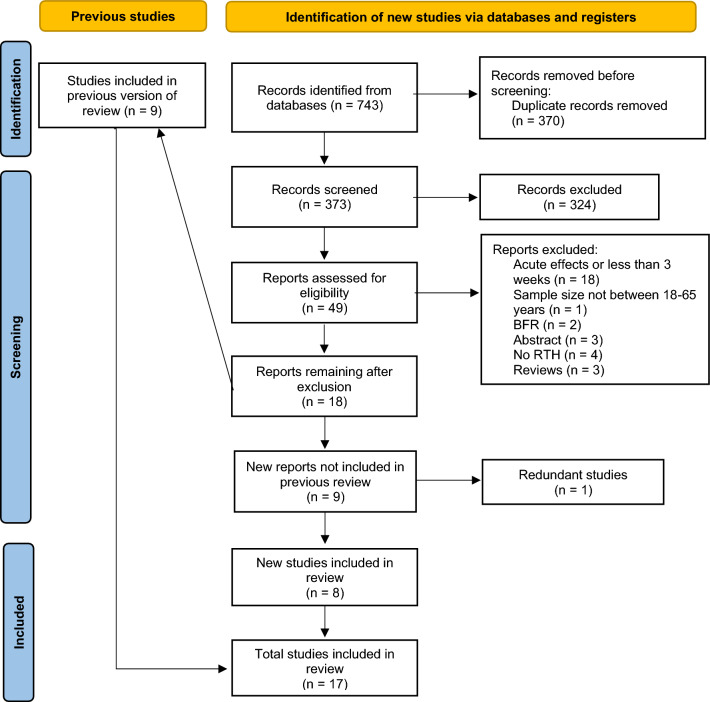
Flow diagram of the search and selection of studies.

The systematic search returned 743 studies. After removal of duplicates and screening by title and abstract, 49 full-text documents were selected for possible inclusion in the meta-analysis. Two of these studies^37,38^ were found to be redundant since they came from the same experiment-i.e., the Manimmanakorn et al.^8^ and Martínez-Guardado et al.^39^ studies, respectively, provided the same data. Another study was subsequently included because it reported additional muscle thickness data^38^. Ultimately, 17 studies (9 from the previous meta-analysis and 8 new studies) met the pre-determined inclusion criteria. A PRISMA flowchart of the search process is shown in Fig. 1.

### Study characteristics

General characteristics of the included studies are summarised in Table 1. The total sample comprised 348 participants (n = 164 for RTN and n = 184 for RTH). These 17 studies assessed changes in muscle hypertrophy (n = 83 for CSA^3,5,8^; n = 184 for lean mass^7,40,41^ and n = 60 for muscle thickness^6,38,42^) and/or strength development (n = 232 for 1RM^15,39,43,44^).

Three studies reported different rest intervals between sets and exercises^8,38,45^ and 1 did not specify the inter-set rest interval^43^; thus, all were excluded from the subanalysis of the inter-set rest factor. One study displayed 2 different levels of hypoxia (severe and moderate)^44^ and both hypoxia comparisons to normoxia were included in the subanalysis of the severity of the hypoxia.

All included studies employed a *live low-train high* (live in normoxia-train in hypoxia) strategy, were published between 2003 and 2022, and had sample sizes ranging from 12 to 37 participants. The mean age of participants ranged from 20.2 ± 3.3^8^ to 29.0 ± 5.0^41^years old, with body weights ranging from 60.2 ± 16^6^ to 98.7 ± 12.8 kg^43^ (Table 1). Only 2 studies totally^8^ or partially^7^ included women in their samples. The hypoxic condition was simulated in all studies. The training status of the sample varied widely across studies with inconsistent terminologies ranging from untrained, recreational, recreationally trained, recreationally strength trained, trained and strength trained to well-trained in a sport discipline and professional context. Given the ambiguity in terminology, we were unable to subanalyze the potential effects of training status on outcomes. Exercise program periods ranged from 3^43^ to 8 weeks^6,38,45,46^ with a mean training frequency of 2–4 sessions per week. Seven studies used lower limb exercises^3,8,15,21,42,44,47^, 2 studies used single-joint arm flexion and extension exercises^5,6^, 3 studies employed a combination of multi-joint upper and lower body exercises^40,41,46^, and 5 studies used a full-body routine^7,38,39,43,45^.

Nine studies were conducted at moderate hypoxia^5,15,21,38,41,43–46^ and 9 at severe hypoxia^3,6–8,39,40,42,44,47^. Four studies employed the use of low-loads (20–50% of 1 RM)^3,7,8,47^, and 3 implemented heavy-load training programs (> 80% of 1 RM)^38,43,45^; the remainder of the studies employed moderate-load programs (60–80% 1RM)^15,41,44^. Seven studies used short inter-set rest intervals (< 60 s)^3,5–8,44,47^, 3 used moderate inter-set rest intervals (> 60–< 120 s)^39,41,46^ and 4 used long inter-set rest intervals (≥ 120 s)^5,15,21,40,42^. (Table 1).

### Meta-analyses

#### Effect of RTH on muscle hypertrophy

**Figure 2:**
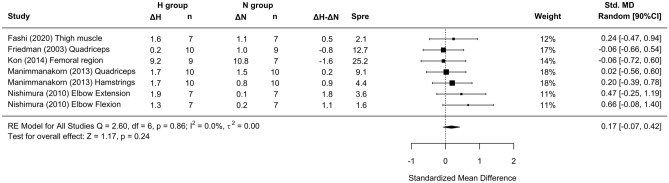
Forest plot of the standardized mean differences of the total effect of the resistance training program between-conditions (hypoxic [H] group vs. normoxic [N] group) on CSA. Δ: mean differences between post–pre in H and N or between H–N; n: sample size; Spre: mean baseline standard deviation; Std. MD: standard mean difference; RE: random effect’s model; CI; confidence interval; Q: test statistic for the test of heterogeneity; df: degrees of freedom; *p*: *p* value; I^2^: I^2^ test; τ^2^: tau^2^ test; Z: z value.

**Figure 3:**
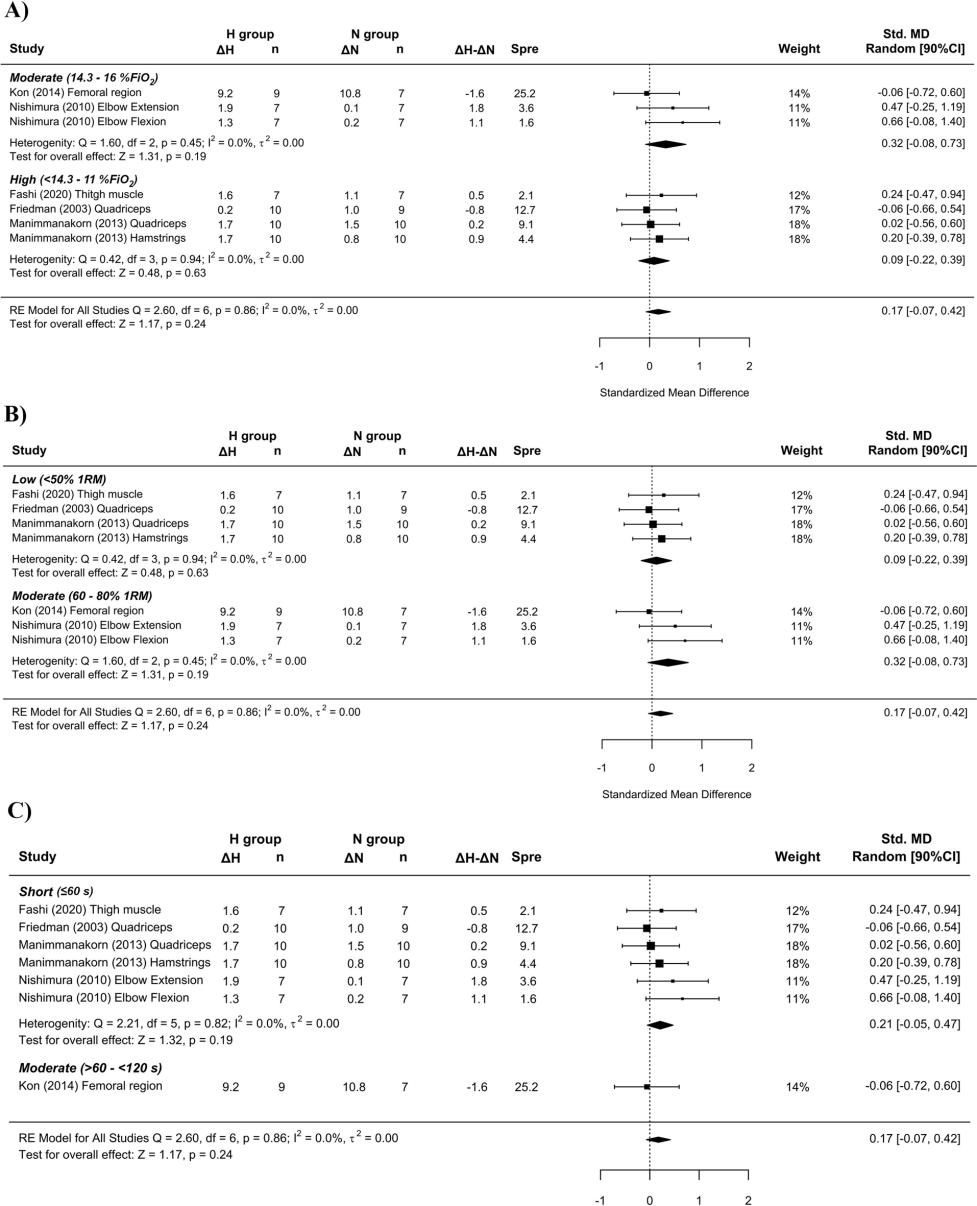
Forest plot of the standardized mean differences of the resistance training program between-conditions (hypoxic [H] group vs. normoxic [N] group) on CSA, subanalysed by: (**A**) severity of the hypoxia; (**B**) training load; and (**C**) interset rest interval. Δ: mean differences between post–pre in H and N or between H-N; n: sample size; Spre: mean baseline standard deviation; Std. MD: standard mean difference; RE: random effect’s model; CI; confidence interval; FiO_2_: fraction of inspired oxygen; 1RM; 1 repetition maximum; Q: test statistic for the test of heterogeneity; df: degrees of freedom; *p*: *p* value; I^2^: I^2^ test; τ^2^: tau^2^ test; Z: z value.

In the basic analysis, trivial differences in CSA favored RTH over RTN conditions (SMD = 0.17 [− 0.07; 0.42]; Fig. 2). Subanalysis indicated a small effect on CSA benefiting RTH with the use of moderate hypoxia (SMD = 0.32 [− 0.08, 0.73]; Fig. 3A) and moderate loads (SMD = 0.32 [− 0.08, 0.73]; Fig. 3B) and a small effect for short inter-set rest intervals (SMD = 0.21 [− 0.05; 0.47]; Fig. 3C).

**Figure 4:**
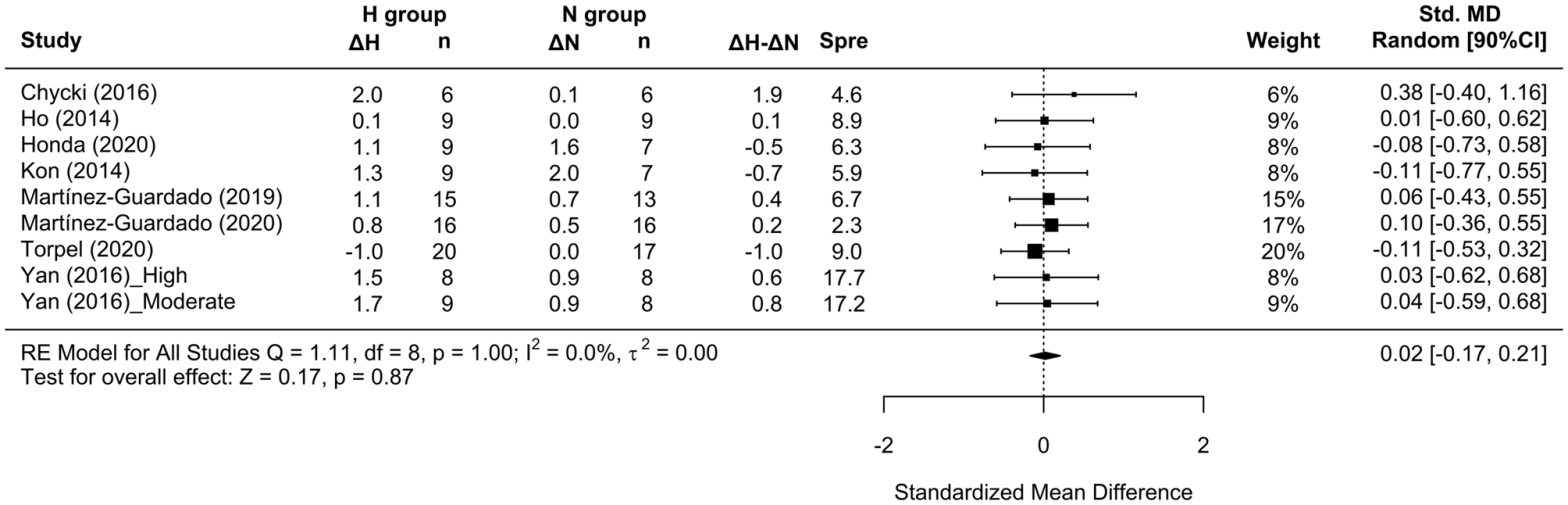
Forest plot of the standardized mean differences of the total effect of the resistance training program between-conditions (hypoxic [H] group vs. normoxic [N] group) on lean mass. Δ: mean differences between post–pre in H and N or between H–N; n: sample size; Spre: mean baseline standard deviation; Std. MD: standard mean difference; RE: random effect’s model; CI; confidence interval; Q: test statistic for the test of heterogeneity; df: degrees of freedom; *p*: *p* value; I^2^: I^2^ test; τ^2^: tau^2^ test; Z: z value. Yan et al.^44^ study provides a group with moderate hypoxia and another with high hypoxia.

**Figure 5:**
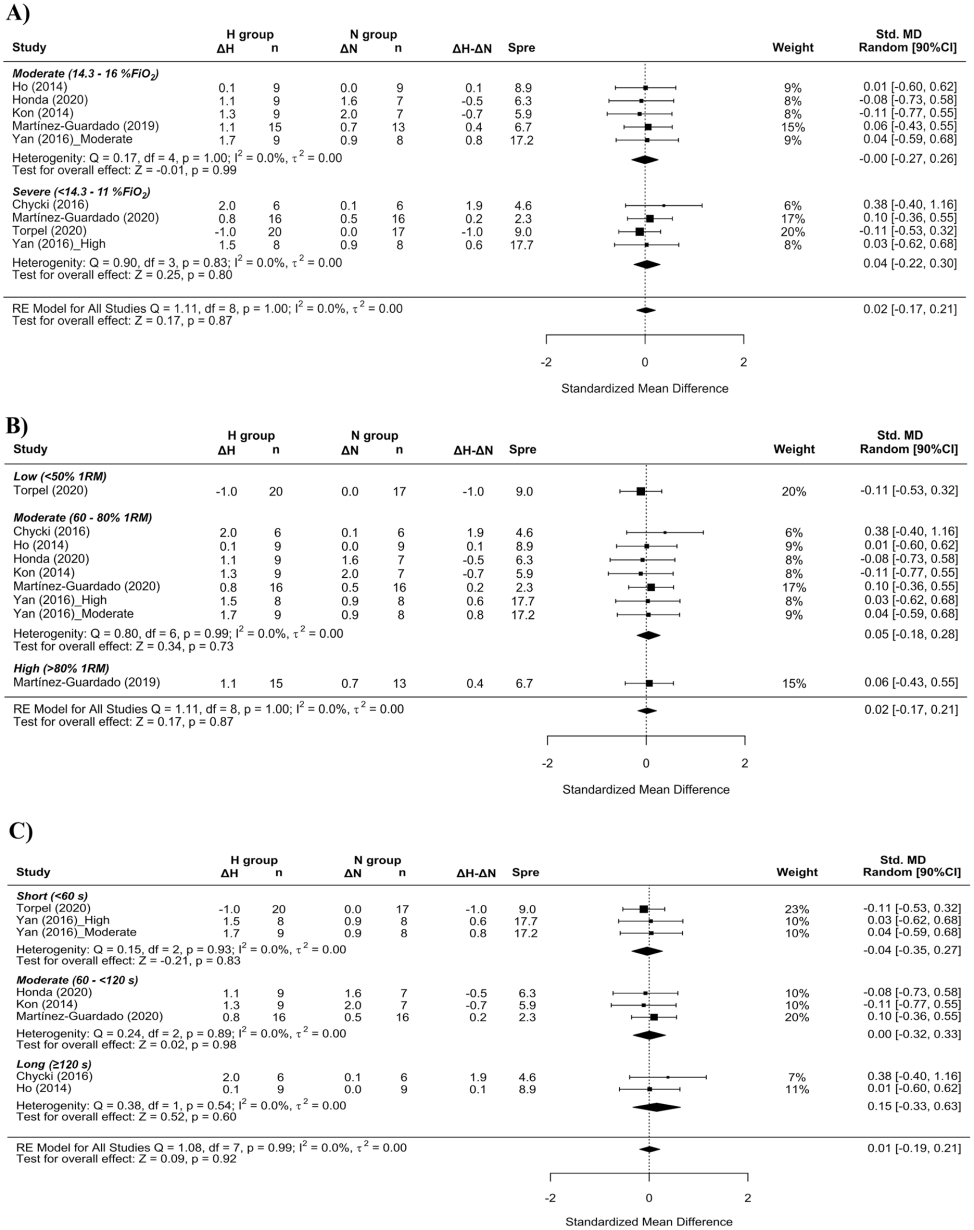
Forest plot of the standardized mean differences of the resistance training program between-conditions (hypoxic [H] group vs. normoxic [N] group) on lean mass subanalysed by: (**A**) severity of the hypoxia; (**B**) training load; and (**C**) interset rest interval. Δ: mean differences between post–pre in H and N or between H–N; n: sample size; Spre: mean baseline standard deviation; Std. MD: standard mean difference; RE: random effect’s model; CI; confidence interval; FiO_2_: fraction of inspired oxygen; 1RM; 1 repetition maximum; Q: test statistic for the test of heterogeneity; df: degrees of freedom; *p*: *p* value; I^2^: I^2^ test; τ^2^: tau^2^ test; Z: z value. Yan et al.^44^ study provides a group with moderate hypoxia and another with high hypoxia.

**Figure 6:**
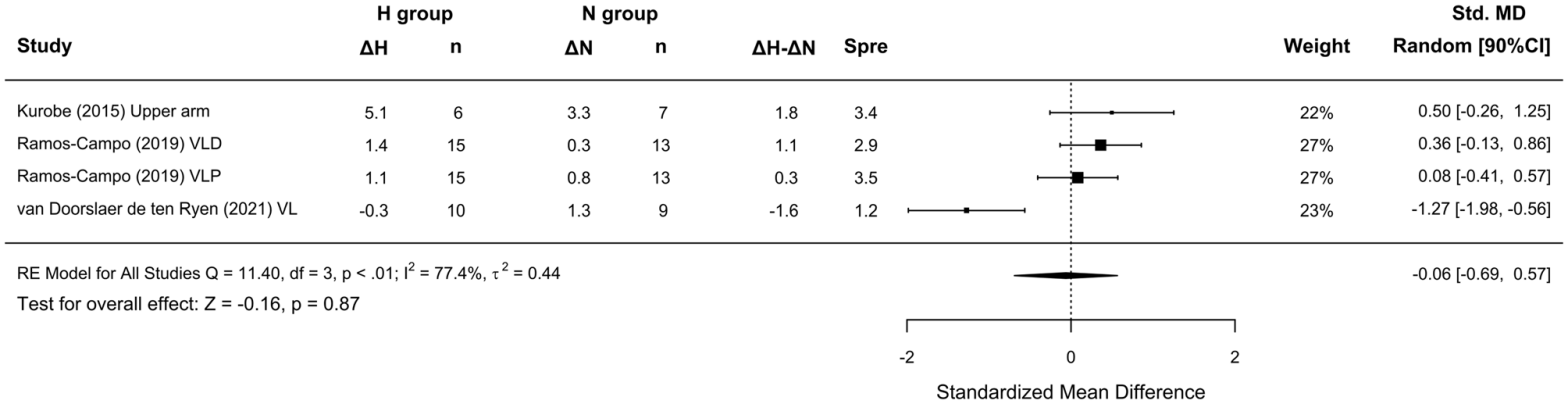
Forest plot of the standardized mean differences of the total effect of the resistance training program between-conditions (hypoxic [H] group vs. normoxic [N] group) on muscle thickness. Δ: mean differences between post–pre in H and N or between H–N; n: sample size; Spre: mean baseline standard deviation; Std. MD: standard mean difference; Random: random effect’s model; CI; confidence interval; Q: test statistic for the test of heterogeneity; df: degrees of freedom; *p*: *p* value; I^2^: I^2^ test; τ^2^: tau^2^ test; Z: z value; VL: vastus lateralis; VLD: vastus lateralis distal; VLP: vastus lateralis proximal.

No differences in lean mass were detected between RTH and RTN (SMD = 0.02 [− 0.17 to 0.21]; Fig. 4); subanalyses did not indicate any effects of the studied covariates (Fig. 5A,B,C). No differences in muscle thickness were observed between environmental conditions (SMD = − 0.06 [− 0.69; 0.57]); there were an insufficient number of studies to carry out subanalyses on this variable (Fig. 6).

Heterogeneity between studies was found to be low for CSA and lean mass (I^2^ = 0%), and high (I^2^ = 77.4%) for muscle thickness.

#### Effect of RTH on strength development

**Figure 7:**
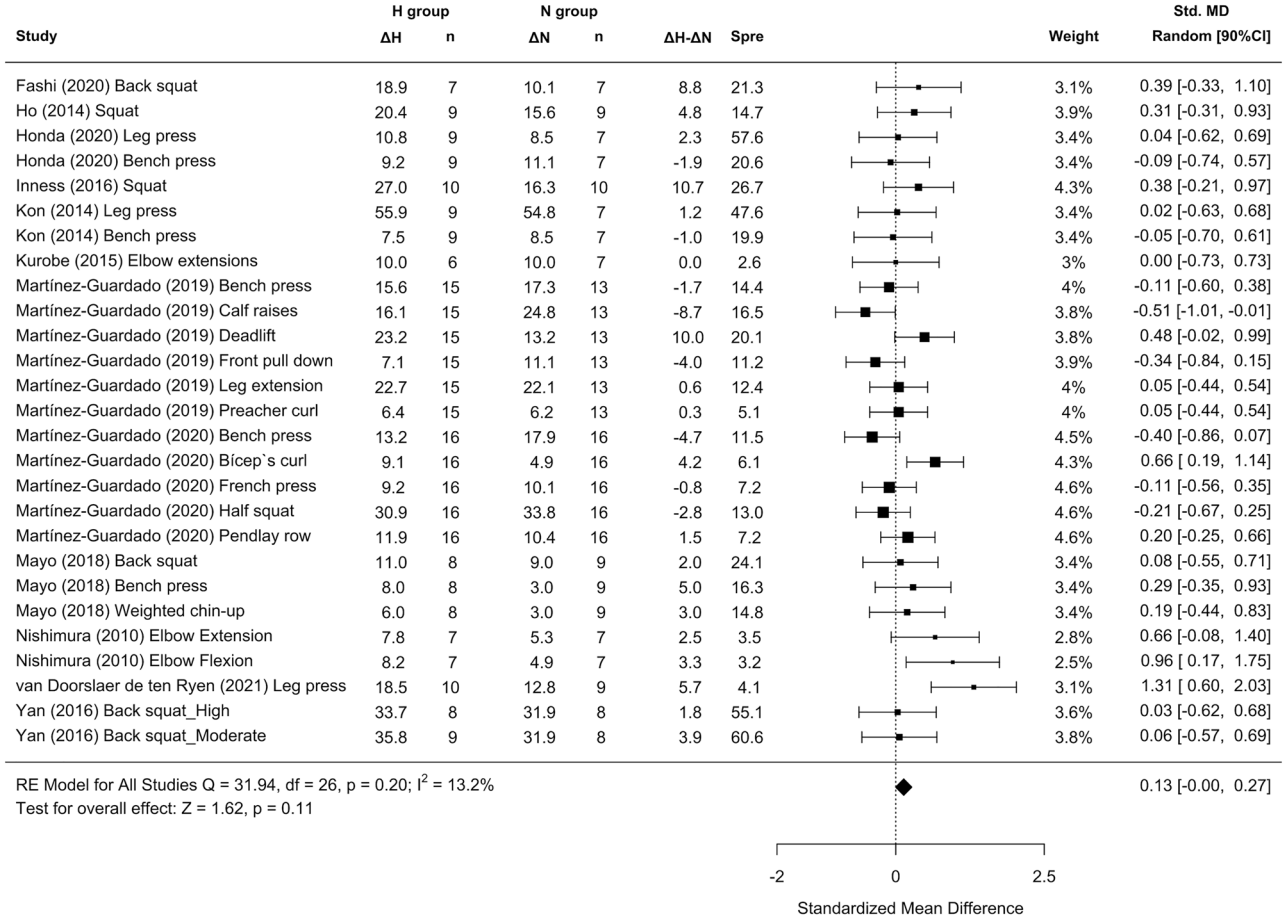
Forest plot of the standardized mean differences of the total effect of the resistance training program between-conditions (hypoxic [H] group vs. normoxic [N] group) on RM. Δ: mean differences between post–pre in H and N or between H–N; n: sample size; Spre: mean baseline standard deviation; Std. MD: standard mean difference; RE: random effect’s model; CI; confidence interval; Q: test statistic for the test of heterogeneity; df: degrees of freedom; *p*: *p* value; I^2^: I^2^ test; Z: z value. Yan et al.^44^ study provides a group with moderate hypoxia and another with high hypoxia.

**Figure 8:**
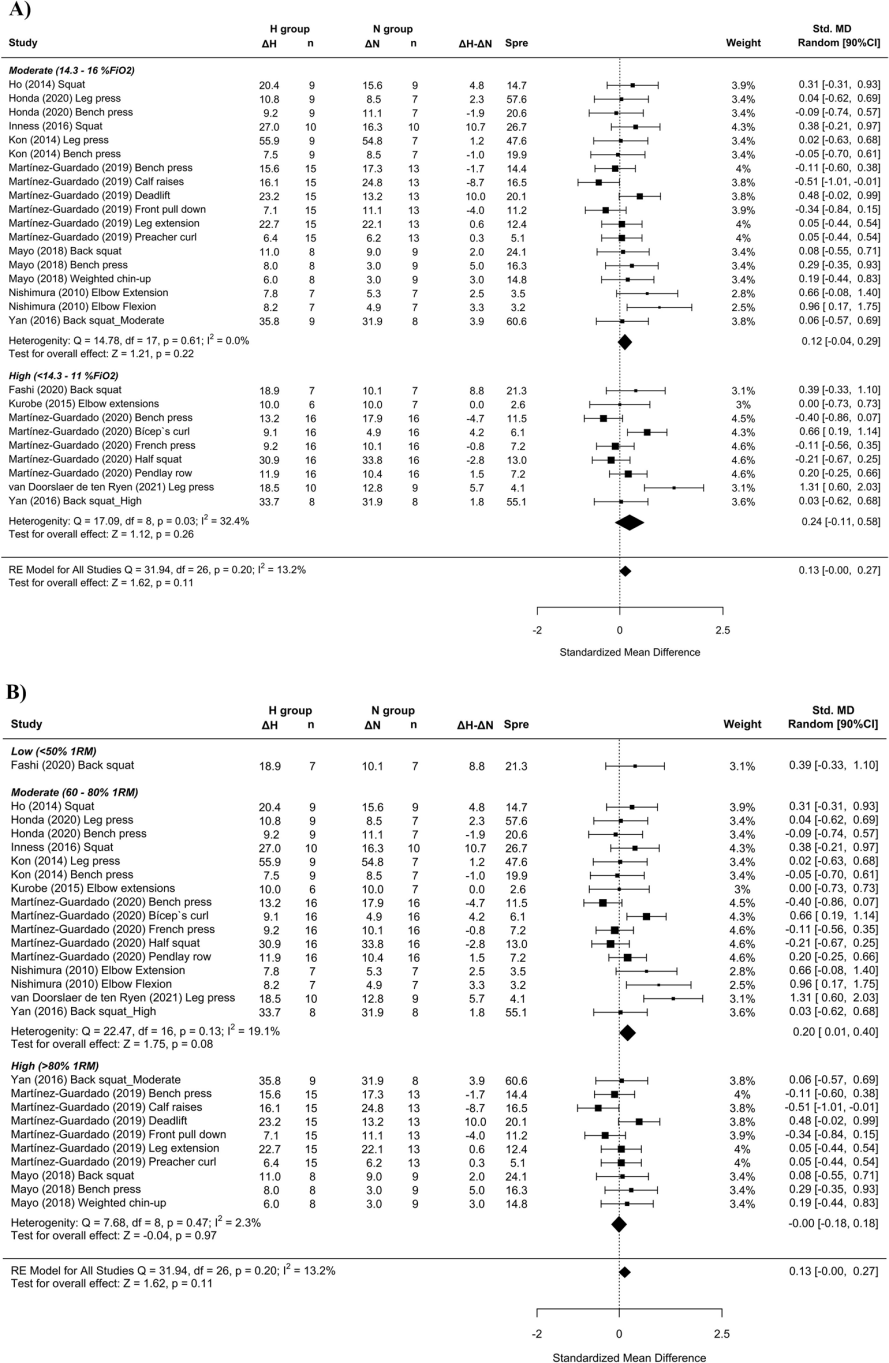

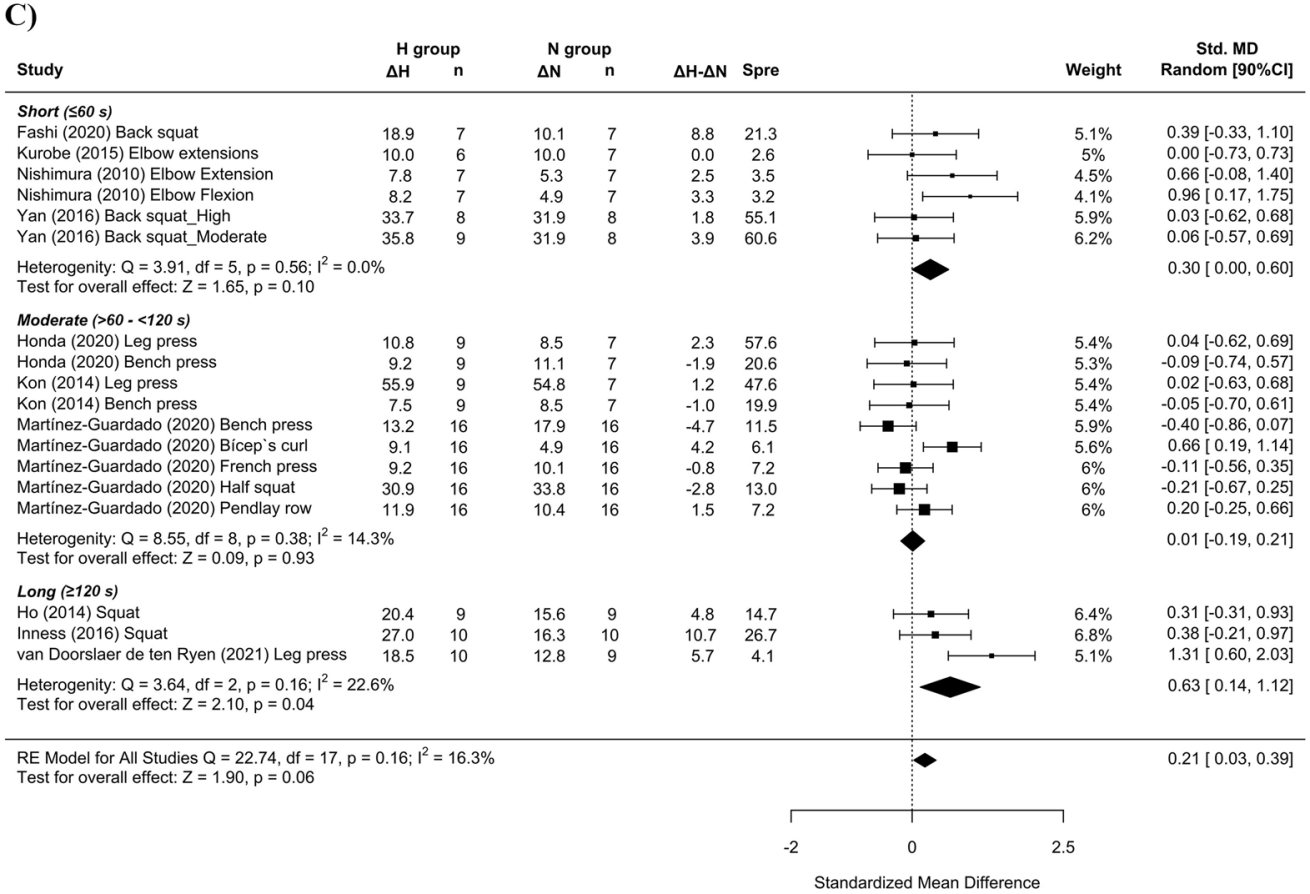
Forest plot of the standardized mean differences of the resistance training program between-conditions (hypoxic [H] group vs. normoxic [N] group) on RM, subanalysed by: (**A**) severity of the hypoxia; (**B**) training load; and (**C**) interset rest interval. Δ: mean differences between post–pre in H and N or between H-N; n: sample size; Spre: mean baseline standard deviation; Std. MD: standard mean difference; RE: random effect’s model; CI; confidence interval; FiO_2_: fraction of inspired oxygen; 1RM; 1 repetition maximum; Q: test statistic for the test of heterogeneity; df: degrees of freedom; *p*: *p* value; I^2^: I^2^ test; Z: z value. Yan et al.^44^ study provides a group with moderate hypoxia and another with high hypoxia.

Twelve studies examined the effect of RTH on strength development. Trivial differences in maximal strength favoring RTH over RTN conditions (SMD = 0.11 [− 0.01; 0.23]; Fig. 7). Subanalysis of the length of the inter-set rest interval showed a medium effect favoring RTH with the use of longer inter-set rest intervals (SMD = 0.63 [0.14; 1.12]; Fig. 8C). A trivial effect was observed favoring RTH with the use of moderate loads (SMD = 0.20 [0.01, 0.40]; Fig. 8B) and severe hypoxia (SMD = 0.24 [− 0.11, 0.58]; Fig. 8A).

Heterogeneity between studies was found to be low for 1RM between environmental conditions (I^2^ = 13.2%).

#### Risk of bias assessment

The risk of bias/methodological quality of primary studies ranged from medium to high quality (mean quality scores = 5.1/5.3) (Table S4). The risk of bias was unclear for concealment of participant randomization and the blinding of outcome data. All the included studies in this meta-analysis indicated a random sequence generation but did not describe the method used. Egger's test suggested a risk of small study bias for muscle strength outcomes (*p* = 0.001); no risk of small study bias was apparent in regard to muscle hypertrophy outcomes (Egger’s test: *p* > 0.05).

## Discussion

This systematic review and meta-analysis synthesised and quantified data on studies that directly compared the effects of R_T_ in hypoxia vs normoxia on muscle hypertrophy and strength development. Our analysis included 17 studies, almost twice as many as in previous reviews on the topic^7,38,39,41–43,45,47^. The inclusion of more recent research (8 additional studies) provided the ability to draw stronger conclusions about the use of RTH for enhancing muscular adaptations.

Similar to previous findings^10,16,17^, a simple pooled analysis without controlling for covariates did not provide compelling support for a potential benefit of RTH versus RTN on muscle hypertrophy (see Figs. 2, 4 and 6) and strength development (see Fig. 7). However, subanalyses of data, which considered previously identified potential biases (training load, inter-set rest interval and severity of the hypoxia), suggest a trivial to medium advantage in the use of moderate training loads and short to longer inter-set rest period in R_T_ at moderate hypoxia on muscular adaptations (see Figs. 3, 5 and 8). However, other potential confounding factors, such as training status or the type of exercise (one or several exercises, monoarticular or polyarticular, large or small muscle groups, among others), could not be subanalysed due to a lack of sufficient representative data. In this regard, our findings indicate that the use of moderate training loads and longer inter-set rest intervals show a trivial to medium beneficial effect of RTH on strength development compared to the same training protocol under normoxic conditions. However, the inter-set rest period impact on CSA was highly influenced by the fact that only one study employed rest intervals longer than 60s^46^; thus, this finding should be interpreted with some caution. Moreover, the use of moderate loads and hypoxia showed a small beneficial effect on CSA compared to low loads and severe hypoxia. Alternatively, changes in lean mass and muscle thickness changes were similar between normoxia and hypoxia irrespective of covariates.

### Effect of RTH on muscle hypertrophy

R_T_ is purported to induce muscle hypertrophy through mechanical, metabolic and hormonal processes^48^. The use of multiple sets of moderate loads and relatively short inter-set rest intervals (60–120 s) between sets has been shown to maximize metabolic stress during R_T_^12^. Accordingly, the use of an intermittent hypoxic environment (moderate or severe) during R_T_ conceivably may elicit a heightened anabolic response compared to normoxic conditions due to the greater accumulation of metabolic byproducts^12,49,50^. Contrarily, evidence shows that chronic exposure (> 3 days) to severe hypoxia could contribute to the loss of muscle mass^51^. To date, there is no longitudinal research on the effect of strength training performed in intermittent or chronic hypoxia at moderate terrestrial altitude. All the studies included in this meta-analysis were carried out in normobaric systemic hypoxia.

A pooled analysis of all studies did not show a beneficial effect for RTH on muscle hypertrophy compared to equivalent training in normoxia (SMD < 0.17). Subanalysis of studies indicated that the use of shorter inter-set rest intervals with RTH had a small benefit on CSA changes (SMD = 0.21 [− 0.05; 0.47]). However, longer inter-set rest periods (> 120 s) also are proposed to extend the capacity to maintain intensities of load and volume during training^49,52^, which in turn could supersede any potential benefits of metabolic stress on hypertrophic adaptations. The paucity of studies with moderate and long inter-set rest intervals in this meta-analysis clouds interpretation of the interaction between environmental conditions and rest period length. Further research is needed to elucidate the underlying mechanisms.

The relatively low number of included studies that assessed lean mass^7,21,39–41,44–46^ and muscle thickness^6,38,42^ did not allow us to draw strong conclusions on these outcomes. No appreciable effects were observed between RTH and RTN (SMD = 0.02 [CI –0.17, 0.21]) in regard to lean mass. Bioelectrical impedance was used in 3 of the studies^7,21,39^, while the other 4 employed dual-energy x-ray absorptiometry^40,41,44,46^; these methods may lack the ability to detect subtle changes in muscle mass^53^. Finally, differences in the composition of exercises in the training protocols (i.e., focused to a specific muscle, body region or full-body) may have also influenced the interpretation of the changes in lean mass. In particular, 3 of the included studies employed full-body routines^7,39,45^, 3 employed 1 exercise for each body region^40,41,46^ and only 2 used a single compound leg exercise (back squat)^21,44^.

Muscle thickness was the least-used method for estimating changes in muscle size in RTH. Among the 3 available studies on this outcome, only one^42^ found a detrimental effect of RTH under severe hypoxia in untrained participants (13.5% F_i_O_2_). Conversely, Kurobe et al.^6^ and Ramos-Campo et al.^38^ reported no significant changes between conditions under severe and moderate hypoxia in untrained and trained populations, respectively. Additionally, differences in the training loads and inter-set rest intervals used among studies compromised the statistical power of the meta-analysis and thus made it difficult to draw strong conclusions about the effect of RTH on muscle thickness.

### Effect of RTH on strength development

The maximum muscular strength was evaluated via the pre-post study change in 1RM. Pooled analysis of all studies did not indicate that RTH increased maximal strength to a greater magnitude than the same training under normoxia; although the direction of the interaction favored RTH, the point estimate indicated minimal benefits on this outcome (SMD = 0.13 [− 0.00; 0.27]). Conversely, subgroup analysis of the data identified long inter-set rest intervals (SMD = 0.63 [0.14; 1.12]) and moderate loads (SMD = 0.20 [0.01; 0.40]) as positive modulators of strength development in RTH, regardless of the severity of hypoxia. Hypoxic conditions varied considerably between studies (ranging from 12 to 16% FiO_2_) and did not seem to meaningfully influence 1RM outcomes; this finding is in opposition with that of hypertrophy, where moderate hypoxia showed a favorable effect on CSA increases. The underlying mechanisms for the observed discrepancy are not readily apparent and warrant future investigation.

The potential of RTH to improve muscle strength is thought to be largely mediated by hypertrophic adaptations (Scott et al., 2014). Hypoxia-mediated neural adaptations, generally linked to the use of heavy loads (> 85% 1RM)^54^, remain poorly elucidated. Nevertheless, as previously mentioned, the observed benefit of moderate loads during hypoxic training could be limited by inconsistencies between experimental designs and participant training level. In this regard, differences in the frequency of weekly sessions and duration of the training protocols confound the ability to draw strong inferences, since neural alterations start during the early phase of training in untrained subjects^3,55^. Finally, it could be argued that hypoxia per se may not confer a favorable environment for neural adaptations in strength training compared to RTN. This hypothesis should be further investigated in future research.

The body of research that included training protocols specific to muscular strength improvements (e.g., higher loads with longer inter-set rests) did not show a benefit to conditions of systemic hypoxia^50^. Only 1 of the 3 included studies that employed long inter-set rest intervals^42^ showed a clear benefit of hypoxia for strength development (Fig. 8C), which only would partially support this beneficial effect. Indeed, recovery periods ≥ 120 s seem to mitigate any additive benefit from the hypoxic stimulus^50^, while shorter inter-set rest periods could entail more challenging metabolic conditions for muscle development^56^. In contrast, our results revealed that longer rest periods produced moderate gains in 1RM after RTH compared to RTN (SMD = 0.63 [0.14; 1.12]). This discrepancy may be due to the use of untrained samples in 2 of the studies^21,42^. Grgic et al.^57^ proposed the use of inter-set rest intervals > 120 s in trained subjects and from < 60 to < 120 s in untrained individuals to maximize gains in muscular strength in normoxia. Hence, the results of our meta-analysis could be influenced by the fact we were not able to subanalyse data based on the participants’ training status.

### Limitations

This meta-analysis has some limitations. First, although most of the studies assessed muscle strength outcomes, several did not report a measurement of muscle hypertrophy. Second, no data from RTH at terrestrial altitudes, and consequently from chronic exposures, were available. Third, results were potentially affected by the divergent methodologies employed in RTH studies, particularly the training protocols and participants’ training status, which could not be analysed as a covariate. Fourth, the small number of studies included in some of the analyses could limit the ability to draw inferences from the obtained outcomes and represent a potential risk of bias.

## Conclusions

Consistent with previous reviews, the overall pooled results remained inconclusive as to the use of RTH compared to RTN for muscular adaptations, despite the inclusion of a substantial body of new research on the topic^58^.

## Practical application

The findings of this systematic review and meta-analysis provide insights for the prescription of resistance training at intermittent systemic hypoxia exposure to promote muscular hypertrophy and strength development. The subgroup analysis revealed 2 conditions under which the use of R_T_ in hypoxia may be of benefit: 1) training programs that employ loads between 60–80% 1RM, inter-set rest intervals of ≤ 60 s and moderate hypoxia show greater increases in muscle CSA; 2) training programs that employ loads between 60–80%1RM and inter-set rest intervals ≥ 120 s show greater increases in strength; however, the severity of hypoxia does not appear relevant to gains in 1RM.

## Future research

We recommend that future research endeavors: (a) promote greater standardization of training protocols that better reflect applicability to participant’ training status; and (b) explore the effect of R_T_ under continuous or intermittent exposure to terrestrial hypoxia, whose physiological responses differs from breathing O_2_-depleted air (normobaric hypoxia)^58^.

### Supplementary Information

Supplementary Information.

## Data Availability

The datasets used and analyzed during the current study are available from the corresponding author on reasonable request.
