# Author Correction: Efficacy of resistance training in hypoxia on muscle hypertrophy and strength development: a systematic review with meta-analysis

**DOI:** 10.1038/s41598-023-42611-2

**Published:** 2023-09-20

**Authors:** Cristina Benavente, Brad J. Schoenfeld, Paulino Padial, Belén Feriche

**Affiliations:** 1 Department of Physical Education and Sport, Faculty of Sport Sciences, University of Granada, Granada, Spain; 2 Department of Exercise Science and Recreation, CUNY Lehman College, The Bronx, NY USA

Correction to: *Scientific Reports* https://doi.org/10.1038/s41598-023-30808-4, published online 04 March 2023

The original version of this Article contained two errors in the meta-analysis. Nishimura et al*.* (5) that employed 1 min inter-set rest intervals between sets and 3 min rest between exercises, was incorrectly categorized in the meta-analysis as long instead of a short rest interval study. Furthermore, Fashi & Ahmadizad (47) used a training load of 50% of the 70%1RM and was incorrectly categorized as moderate training load instead of low training load.

Authors re-analyzed the data using the correct classifications of these studies and as a result, the Abstract, Methods, Results and Discussion have been revised.

In the Abstract,

“Subanalyses indicated a medium effect on CSA for longer inter-set rest intervals and a small effect for moderate hypoxia and moderate loads favoring RTH. Moreover, a moderate effect for longer inter-set rest intervals and a trivial effect for severe hypoxia and moderate loads favoring RTH was found on 1RM. Evidence suggests that RTH employed with moderate loads (60–80% 1RM) and short (≤ 60 s) to longer inter-set rest intervals (≥ 120 s) respectively enhances muscle hypertrophy and strength compared to normoxia.”

now reads:

“Subanalyses indicated a small effect on CSA for shorter inter-set rest intervals, moderate hypoxia and moderate loads favoring RTH. Moreover, a medium effect for longer inter-set rest intervals and a trivial to small effect for severe hypoxia and moderate loads favoring RTH was found on 1RM. Evidence suggests that RTH employed with moderate loads (60–80% 1RM) enhances both hypertrophy and strength. Hypertrophy appears to benefit from shorter (≤ 60 s) inter-set rest intervals during RTH while greater gains in strength are achieved with longer rest intervals (≥ 120 s).”

In the Method section, under the subheading ‘Data extraction and study outcomes’,

In Table 1, the methodology for Fashi and Ahmadizad and Nishimura et al. were incorrect. The correct and incorrect methodologies appear below.

Incorrect:

**Table:** 

Study	n	H condition	Effective FiO_2_	Training level	Age (years)	Weight (Kg)	Training intervention			Variables measured	
							Weeks (s/w)	Methodology	Exercise	Strength development	Muscle hypertrophy
Fashi and Ahmadizad^47^	7 (M)	NH (gas)	12,7% FiO_2_	Untrained	21 ± 4		4 (3)	3 sets x reps. to failure. 50% 10RM (75% 1RM) (Rest 60 s)	Back squat	RM	CSA
	7 (M)	N (gas)	20,9% FiO_2_								
Nishimura et al.^5^	7 (M)	NH (room)	16% FiO_2_	Untrained	22.7 ± 2.7	66.8 ± 6.0	6 (2)	4 sets × 10 reps. 70% RM (Rest 180 s)	Elbow flexion Elbow extension	RM	CSA flexors CSA extensors
	7 (M)	N (outside)	21% FiO_2_		21.6 ± 1.6	65.0 ± 8.1					

Correct:

**Table:** 

Study	n	H condition	Effective FiO_2_	Training level	Age (years)	Weight (kg)	Training intervention			Variables measured	
							Weeks (s/w)	Methodology	Exercise	Strength development	Muscle hypertrophy
Fashi and Ahmadizad^47^	7 (M)	NH (gas)	12.7% FiO_2_	Untrained	21 ± 4		4 (3)	3 sets x reps. to failure. 50% 10RM (~ 37% RM) (Rest 60 s)	Back squat	RM	CSA
	7 (M)	N (gas)	20.9% FiO_2_								
Nishimura et al.^5^	7 (M)	NH (room)	16% FiO_2_	Untrained	22.7 ± 2.7	66.8 ± 6.0	6 (2)	4 sets × 10 reps. 70% RM (Rest 60 s)	Elbow flexion Elbow extension	RM	CSA flexors CSA extensors
	7 (M)	N (outside)	21% FiO_2_		21.6 ± 1.6	65.0 ± 8.1					

In the Results section, under the subheading ‘Study characteristics’,

“Three studies employed the use of low-loads (20–50% of 1 RM)^3,7,8^, and 3 implemented heavy-load training programs (>  80% of 1 RM)^38,43,45^; the remainder of the studies employed moderate-load programs (60–80% 1RM)^15,41,44^.”

now reads:

“Four studies employed the use of low-loads (20–50% of 1 RM)^3,7,8,47^, and 3 implemented heavy-load training programs (>  80% of 1 RM)^38,43,45^; the remainder of the studies employed moderate-load programs (60–80% 1RM)^15,41,44^.^”^

And,

“Six studies used short inter-set rest intervals (< 60 s)^3,6–8,44,47^, 3 used moderate inter-set rest intervals (> 60–< 120 s)^39,41,46^ and 5 used long inter-set rest intervals (≥ 120 s)^5,15,21,40,42^. (Table 1).”

now reads:

“Seven studies used short inter-set rest intervals (≤ 60 s)^3,5–8,44,47^, 3 used moderate inter-set rest intervals (> 60–< 120s)^39,41,46^ and 4 used long inter-set rest intervals (≥ 120 s)^15,21,40,42^. (Table 1).”

Additionally, in the Results section, under the subheading ‘Meta-analyses. Effect of RTH on muscle hypertrophy’,

“Subanalysis indicated a small effect on CSA benefiting RTH with the use of moderate hypoxia (SMD = 0.32 [− 0.08, 0.73]; Figure 3A) and moderate loads (SMD = 0.30 [− 0.05, 0.65]; Figure 3B) and a medium effect for longer inter-set rest intervals (SMD = 0.56 [0.05; 1.08]; Figure 3C).”

now reads:

“Subanalysis indicated a small effect on CSA benefiting RTH with the use of moderate hypoxia (SMD = 0.32 [− 0.08, 0.73]; Figure 3A) and moderate loads (SMD = 0.32 [− 0.08, 0.73]; Figure 3B) and a small effect for short inter-set rest intervals (SMD = 0.21 [− 0.05; 0.47]; Figure 3C).”

Furthermore, in the Results section, under the subheading ‘Meta-analyses. Effect of RTH on strength development’,

“Subanalysis of the length of the inter-set rest interval showed a medium effect favoring RTH with the use of longer inter-set rest intervals (SMD = 0.67 [0.36; 0.98]; Figure 8C). A trivial effect was observed favoring RTH with the use of moderate loads (SMD = 0.18 [0.02, 0.34]; Figure 8B) and severe hypoxia (SMD = 0.16 [− 0.10, 0.43]; Figure 8A).”

now reads:

“Subanalysis of the length of the inter-set rest interval showed a medium effect favoring RTH with the use of longer inter-set rest intervals (SMD = 0.63 [0.14; 1.12]; Figure 8C). A trivial effect was observed favoring RTH with the use of moderate loads (SMD = 0.20 [0.01, 0.40]; Figure 8B) and severe hypoxia (SMD = 0.24 [− 0.11, 0.58]; Figure 8A).”

And,

“Heterogeneity between studies was found to be low for 1RM between environmental conditions (I^2^ = 18.7%)”

now reads:

“Heterogeneity between studies was found to be low for 1RM between environmental conditions (I^2^ = 13.2%).”

In the Figures,

Panels (B) and (C) in Figure 3 and Figure 8 contained errors and have been updated. The legends are not affected. The original Figures 3 and 8 and accompanying legends appear below.

In the Discussion,

“However, subanalyses of data, which considered previously identified potential biases (training load, inter-set rest interval and severity of the hypoxia), suggest a small to medium advantage in the use of moderate training loads and longer inter-set rest period in RT at moderate hypoxia on muscular adaptations (see Fig 3, 5 & 8).”

now reads:

“However, subanalyses of data, which considered previously identified potential biases (training load, inter-set rest interval and severity of the hypoxia), suggest a trivial to medium advantage in the use of moderate training loads and short to longer inter-set rest period in R_T_ at moderate hypoxia on muscular adaptations (see Fig 3, 5 & 8).”

And,

“In this regard, our findings indicate that the use of moderate training loads and longer inter-set rest intervals show a small to medium beneficial effect of RTH on strength development and CSA improvements compared to the same training protocol under normoxic conditions. However, the inter-set rest period impact on CSA was highly influenced by the results of a single study and thus should be interpreted with some caution^5^. Moreover, the use of moderate hypoxia showed a small beneficial effect on CSA compared to severe.”

now reads:

“In this regard, our findings indicate that the use of moderate training loads and longer inter-set rest intervals show a trivial to medium beneficial effect of RTH on strength development compared to the same training protocol under normoxic conditions. However, the inter-set rest period impact on CSA was highly influenced by the fact that only one study employed rest intervals longer than 60 s^46^; thus, this finding should be interpreted with some caution. Moreover, the use of moderate loads and hypoxia showed a small beneficial effect on CSA compared to low loads and severe hypoxia.”

In the Discussion section, under the subheading ‘Effect of RTH on muscle hypertrophy’,

“A pooled analysis of all studies did not show a beneficial effect for RTH on muscle hypertrophy compared to equivalent training in normoxia (SMD < 0.17). Intriguingly, although short inter-set rest intervals (60–120 s) have been shown to maximize metabolite accumulation, subanalysis of studies indicated that the use of longer inter-set rest intervals with RTH had a moderate benefit on CSA changes (SMD = 0.56 [0.05; 1.08]). Longer inter-set rest periods (> 120 s) are proposed to extend the capacity to maintain intensities of load and volume during training^49,52^, which in turn may have superseded any potential benefits of metabolic stress on hypertrophic adaptations. The interaction between environmental conditions and rest period length is not clear and further research is needed to elucidate the underlying mechanisms for this finding.”

now reads:

“A pooled analysis of all studies did not show a beneficial effect for RTH on muscle hypertrophy compared to equivalent training in normoxia (SMD < 0.17). Subanalysis of studies indicated that the use of shorter inter-set rest intervals with RTH had a small benefit on CSA changes (SMD = 0.21 [− 0.05; 0.47]). However, longer inter-set rest periods (> 120 s) also are proposed to extend the capacity to maintain intensities of load and volume during training^49,52^, which in turn could supersede any potential benefits of metabolic stress on hypertrophic adaptations. The paucity of studies with moderate and long inter-set rest intervals in this meta-analysis clouds interpretation of the interaction between environmental conditions and rest period length. Further research is needed to elucidate the underlying mechanisms.”

In the Discussion section, under the subheading ‘Effect of RTH on strength development’,

“Pooled analysis of all studies did not indicate that RTH increased maximal strength to a greater magnitude than the same training under normoxia (p = 0.14); although the direction of the interaction favored RTH, the point estimate indicated minimal benefits on this outcome (SMD = 0.11 [− 0.01; 0.23]). Conversely, subgroup analysis of the data identified long inter-set rest intervals (SMD = 0.67 [0.36;0.98]; p < 0.001) and moderate loads (SMD = 0.18 [0.02; 0.34]; p = 0.07) as positive modulators of strength development in RTH, regardless of the severity of hypoxia.”

now reads:

“Pooled analysis of all studies did not indicate that RTH increased maximal strength to a greater magnitude than the same training under normoxia; although the direction of the interaction favored RTH, the point estimate indicated minimal benefits on this outcome (SMD = 0.13 [− 0.00; 0.27]). Conversely, subgroup analysis of the data identified long inter-set rest intervals (SMD = 0.63 [0.14; 1.12]) and moderate loads (SMD = 0.20 [0.01; 0.40]) as positive modulators of strength development in RTH, regardless of the severity of hypoxia.”

And,

“Only 2 of the 4 included studies that employed long inter-set rest intervals^5,42^ showed a clear benefit of hypoxia for strength development (Figure 8.C), which only would partially support this beneficial effect.”

now reads:

“Only 1 of the 3 included studies that employed long inter-set rest intervals^42^ showed a clear benefit of hypoxia for strength development (Figure 8.C), which only would partially support this beneficial effect.”

And,

“In contrast, our results revealed that longer rest periods produced moderate gains in 1RM after RTH compared to RTN (SMD = 0.67 [0.36; 0.98]; p < 0.001). This discrepancy may be due to the use of untrained samples in 3 of the studies^5,21,42^.”

now reads:

“In contrast, our results revealed that longer rest periods produced moderate gains in 1RM after RTH compared to RTN (SMD = 0.63 [0.14; 1.12]). This discrepancy may be due to the use of untrained samples in 2 of the studies^21,42^.”

Additionally, in the section of ‘Practical Applications’,

“The subgroup analysis revealed 2 conditions under which the use of RT in hypoxia may be of benefit: 1) training programs that employ loads between 60–80%1RM and inter-set rest intervals ≥ 120 s show greater increases in muscle strength and CSA compared to normoxia; and 2) moderate hypoxia seems to be more suitable for improvement in muscle hypertrophy compared to severe hypoxia, while this covariate does not appear relevant to gains in 1RM.”

now reads:

“The subgroup analysis revealed 2 conditions under which the use of RT in hypoxia may be of benefit: 1) training programs that employ loads between 60–80%1RM, inter-set rest intervals of ≤ 60 s and moderate hypoxia show greater increases in muscle CSA; 2) training programs that employ loads between 60–80%1RM and inter-set rest intervals ≥ 120 s show greater increases in strength; however, the severity of hypoxia does not appear relevant to gains in 1RM.”

The original Article has been corrected.

**Figure 3:**
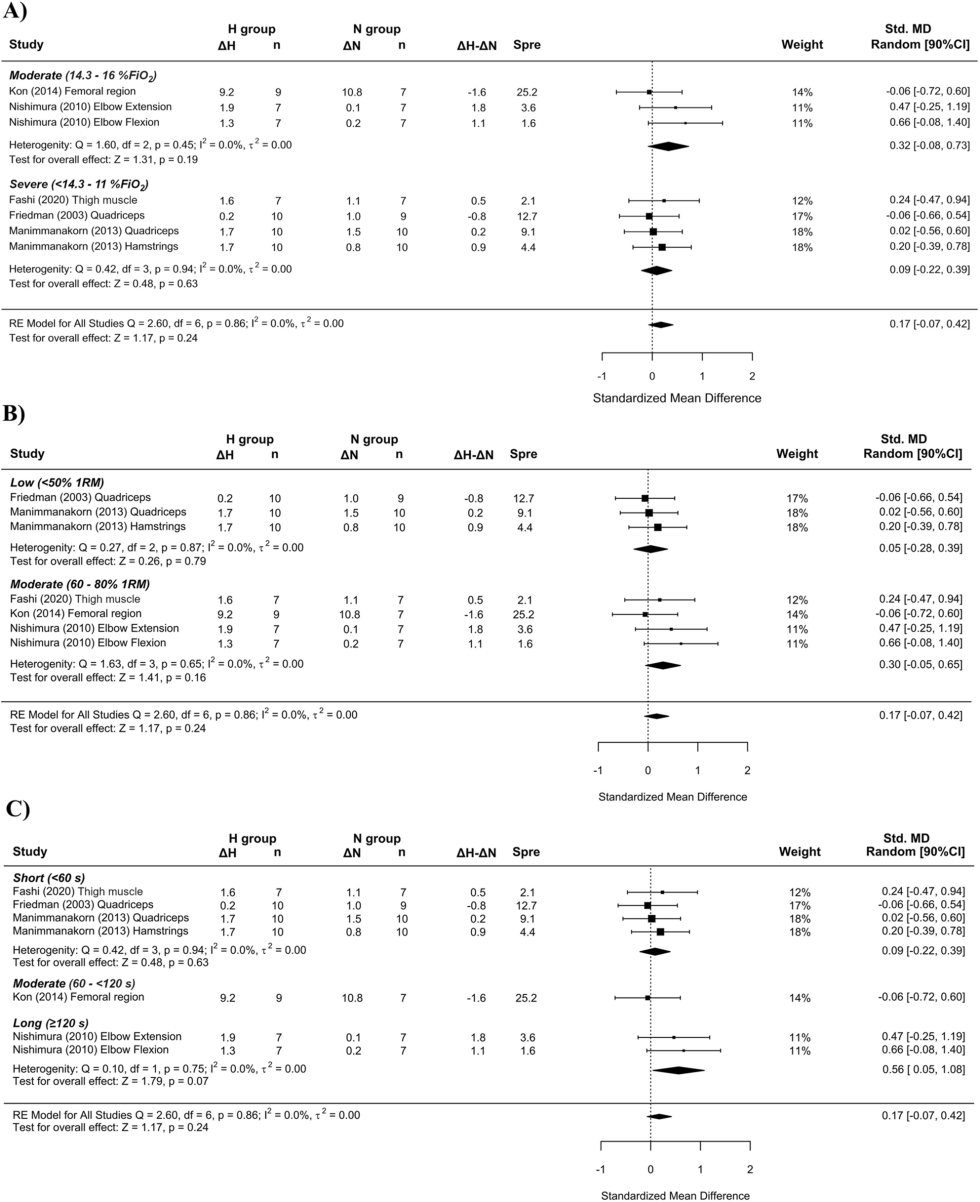
Forest plot of the standardized mean differences of the resistance training program between-conditions (hypoxic [H] group vs. normoxic [N] group) on CSA, subanalysed by: (**A**) severity of the hypoxia; (**B**) training load; and (**C**) interset rest interval. Δ: mean differences between post–pre in H and N or between H-N; n: sample size; Spre: mean baseline standard deviation; Std. MD: standard mean difference; RE: random effect’s model; CI; confidence interval; FiO_2_: fraction of inspired oxygen; 1RM; 1 repetition maximum; Q: test statistic for the test of heterogeneity; df: degrees of freedom; *p*: *p* value; I^2^: I^2^ test; τ^2^: tau^2^ test; Z: z value.

**Figure 8:**
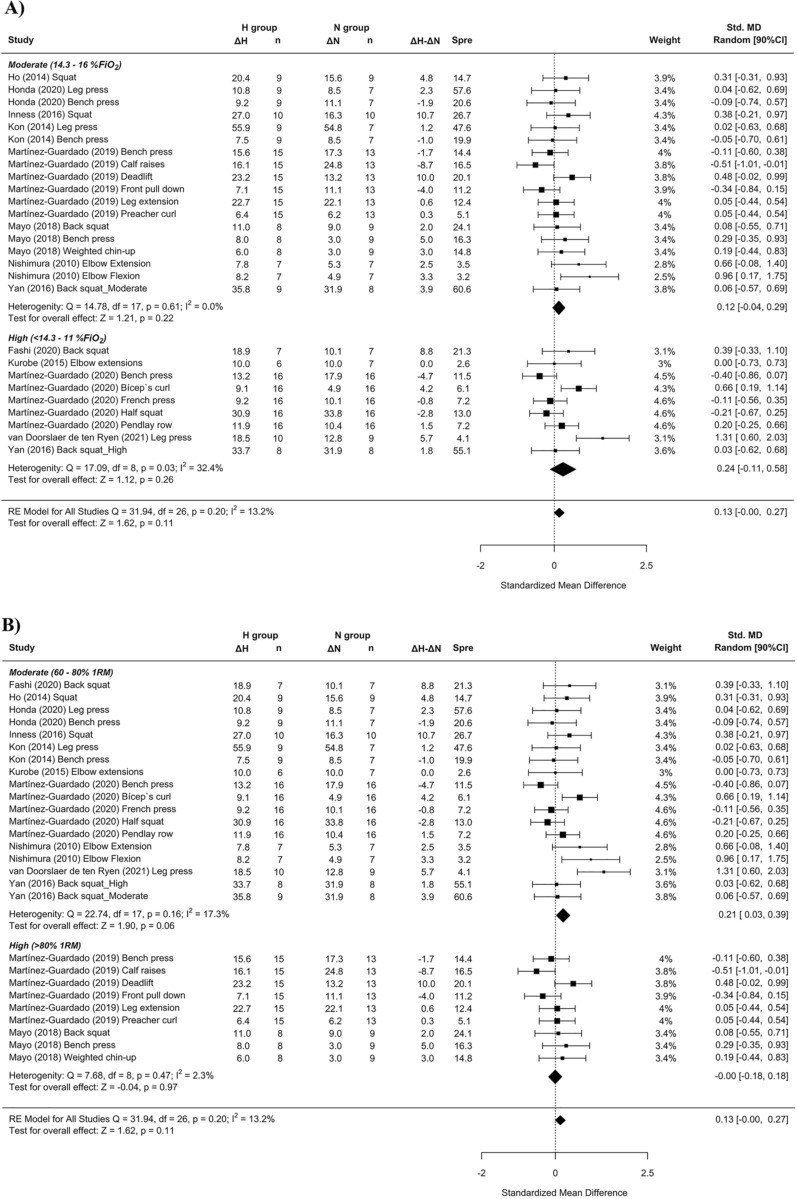

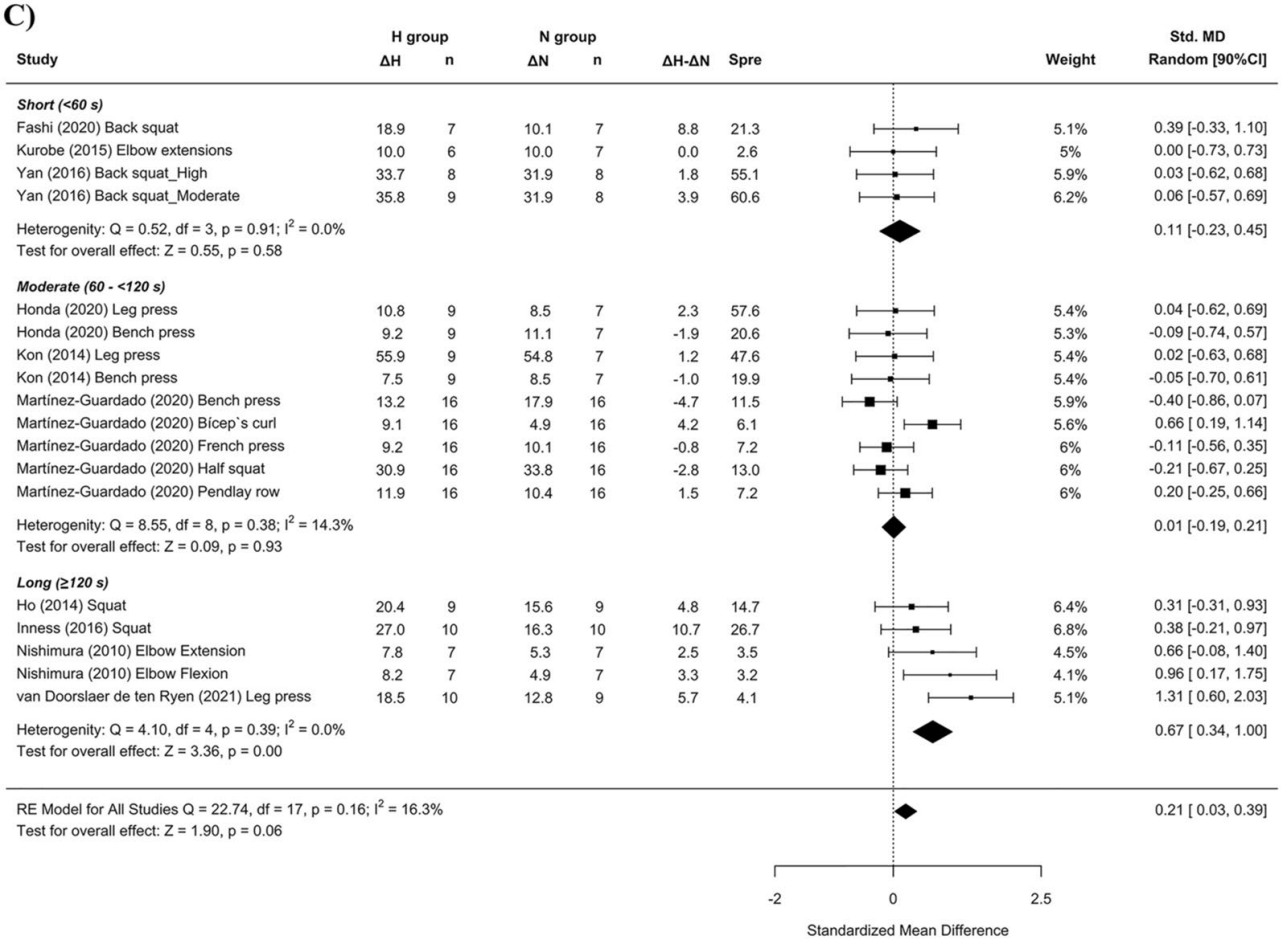
Forest plot of the standardized mean differences of the resistance training program between-conditions (hypoxic [H] group vs. normoxic [N] group) on RM, subanalysed by: (**A**) severity of the hypoxia; (**B**) training load; and (**C**) interset rest interval. Δ: mean differences between post–pre in H and N or between H-N; n: sample size; Spre: mean baseline standard deviation; Std. MD: standard mean difference; RE: random effect’s model; CI; confidence interval; FiO_2_: fraction of inspired oxygen; 1RM; 1 repetition maximum; Q: test statistic for the test of heterogeneity; df: degrees of freedom; *p*: *p* value; I^2^: I^2^ test; Z: z value. Yan et al.^44^ study provides a group with moderate hypoxia and another with high hypoxia.

