# Supplementary material for: Efficacy of resistance training in hypoxia on muscle hypertrophy and strength development: a systematic review with meta-analysis
Source: Sci Rep. 2023 Mar 4;13:3676. doi: 10.1038/s41598-023-30808-4 (PMC9985626; doi:10.1038/s41598-023-30808-4)

Efficacy of resistance training in hypoxia: A systematic review and meta-analysis of effects on strength and hypertrophy.

Cristina Benavente[1]; Brad J. Schoenfeld[2]; Paulino Padial[1]; Belén Feriche[1].

[1]Department of Physical Education and Sport, Faculty of Sport Sciences, University of Granada, Granada, Spain.

[2]Department of Exercise Science and Recreation, CUNY Lehman College, New York, United States of America.

Supplementary information

Table S1. PRISMA for abstract checklist.

Section and Topic	Item #	Checklist item	Reported (Yes/No)
**TITLE**			
Title	1	Identify the report as a systematic review.	Yes
**BACKGROUND**			
Objectives	2	Provide an explicit statement of the main objective(s) or question(s) the review addresses.	Yes
**METHODS**			
Eligibility criteria	3	Specify the inclusion and exclusion criteria for the review.	Yes
Information sources	4	Specify the information sources (e.g. databases, registers) used to identify studies and the date when each was last searched.	Yes
Risk of bias	5	Specify the methods used to assess risk of bias in the included studies.	Yes
Synthesis of results	6	Specify the methods used to present and synthesise results.	Yes
**RESULTS**			
Included studies	7	Give the total number of included studies and participants and summarise relevant characteristics of studies.	Yes
Synthesis of results	8	Present results for main outcomes, preferably indicating the number of included studies and participants for each. If meta-analysis was done, report the summary estimate and confidence/credible interval. If comparing groups, indicate the direction of the effect (i.e. which group is favoured).	Yes
**DISCUSSION**			
Limitations of evidence	9	Provide a brief summary of the limitations of the evidence included in the review (e.g. study risk of bias, inconsistency and imprecision).	Yes
Interpretation	10	Provide a general interpretation of the results and important implications.	Yes
**OTHER**			
Funding	11	Specify the primary source of funding for the review.	Yes
Registration	12	Provide the register name and registration number.	N/A

Table S2. PRISMA checklist.

Section and Topic	Item #	Checklist item	Location where item is reported
**TITLE**			
Title	1	Identify the report as a systematic review.	Title
**ABSTRACT**			
Abstract	2	See the PRISMA 2020 for Abstracts checklist.	Table S1<br>Abstract
**INTRODUCTION**			
Rationale	3	Describe the rationale for the review in the context of existing knowledge.	Pages 3-4
Objectives	4	Provide an explicit statement of the objective(s) or question(s) the review addresses.	Page 4
**METHODS**			
Eligibility criteria	5	Specify the inclusion and exclusion criteria for the review and how studies were grouped for the syntheses.	Pages 5-6
Information sources	6	Specify all databases, registers, websites, organizations, reference lists and other sources searched or consulted to identify studies. Specify the date when each source was last searched or consulted.	Page 5
Search strategy	7	Present the full search strategies for all databases, registers and websites, including any filters and limits used.	Page 5<br>Figure 1
Selection process	8	Specify the methods used to decide whether a study met the inclusion criteria of the review, including how many reviewers screened each record and each report retrieved, whether they worked independently, and if applicable, details of automation tools used in the process.	Pages 5-7<br>Table S3
Data collection process	9	Specify the methods used to collect data from reports, including how many reviewers collected data from each report, whether they worked independently, any processes for obtaining or confirming data from study investigators, and if applicable, details of automation tools used in the process.	Pages 5-7<br>Figure 1
Data items	10a	List and define all outcomes for which data were sought. Specify whether all results that were compatible with each outcome domain in each study were sought (e.g. for all measures, time points, analyses), and if not, the methods used to decide which results to collect.	Table 1
	10b	List and define all other variables for which data were sought (e.g. participant and intervention characteristics, funding sources). Describe any assumptions made about any missing or unclear information.	Table S3<br>Page 5
Study risk of bias assessment	11	Specify the methods used to assess risk of bias in the included studies, including details of the tool(s) used, how many reviewers assessed each study and whether they worked independently, and if applicable, details of automation tools used in the process.	Table S4
Effect measures	12	Specify for each outcome the effect measure(s) (e.g. risk ratio, mean difference) used in the synthesis or presentation of results.	Pages 7-8<br>Table S5
Synthesis methods	13a	Describe the processes used to decide which studies were eligible for each synthesis (e.g. tabulating the study intervention characteristics and comparing against the planned groups for each synthesis (item #5)).	Pages 6-7
	13b	Describe any methods required to prepare the data for presentation or synthesis, such as handling of missing summary statistics, or data conversions.	Pages 6-7
	13c	Describe any methods used to tabulate or visually display results of individual studies and syntheses.	Pages 6-7
	13d	Describe any methods used to synthesize results and provide a rationale for the choice(s). If meta-analysis was performed, describe the model(s), method(s) to identify the presence and extent of statistical heterogeneity, and software package(s) used.	Pages 7-8
	13e	Describe any methods used to explore possible causes of heterogeneity among study results (e.g. subgroup analysis, meta-regression).	Pages 7-8
	13f	Describe any sensitivity analyses conducted to assess robustness of the synthesized results.	Page 8

Section and Topic	Item #	Checklist item	Location where item is reported
Reporting bias assessment	14	Describe any methods used to assess risk of bias due to missing results in a synthesis (arising from reporting biases).	Page 7
Certainty assessment	15	Describe any methods used to assess certainty (or confidence) in the body of evidence for an outcome.	Page 7
**RESULTS**			
Study selection	16a	Describe the results of the search and selection process, from the number of records identified in the search to the number of studies included in the review, ideally using a flow diagram.	Page 9
	16b	Cite studies that might appear to meet the inclusion criteria, but which were excluded, and explain why they were excluded.	Page 9
Study characteristics	17	Cite each included study and present its characteristics.	Pages 9-10
Risk of bias in studies	18	Present assessments of risk of bias for each included study.	Table S4
Results of individual studies	19	For all outcomes, present, for each study: (a) summary statistics for each group (where appropriate) and (b) an effect estimate and its precision (e.g. confidence/credible interval), ideally using structured tables or plots.	Pages 11-12
Results of syntheses	20a	For each synthesis, briefly summarise the characteristics and risk of bias among contributing studies.	Pages 11-12
	20b	Present results of all statistical syntheses conducted. If meta-analysis was done, present for each the summary estimate and its precision (e.g. confidence/credible interval) and measures of statistical heterogeneity. If comparing groups, describe the direction of the effect.	Pages 11-12
	20c	Present results of all investigations of possible causes of heterogeneity among study results.	Pages 11-12
	20d	Present results of all sensitivity analyses conducted to assess the robustness of the synthesized results.	Pages 11-12
Reporting biases	21	Present assessments of risk of bias due to missing results (arising from reporting biases) for each synthesis assessed.	N/A
Certainty of evidence	22	Present assessments of certainty (or confidence) in the body of evidence for each outcome assessed.	Pages 11-12
**DISCUSSION**			
Discussion	23a	Provide a general interpretation of the results in the context of other evidence.	Pages 12-16
	23b	Discuss any limitations of the evidence included in the review.	Pages 16-17
	23c	Discuss any limitations of the review processes used.	Pages 16-17
	23d	Discuss implications of the results for practice, policy, and future research.	Page 17
**OTHER INFORMATION**			
Registration and protocol	24a	Provide registration information for the review, including register name and registration number, or state that the review was not registered.	N/A
	24b	Indicate where the review protocol can be accessed, or state that a protocol was not prepared.	N/A
	24c	Describe and explain any amendments to information provided at registration or in the protocol.	N/A
Support	25	Describe sources of financial or non-financial support for the review, and the role of the funders or sponsors in the review.	Title page
Competing interests	26	Declare any competing interests of review authors.	Title page
Availability of data, code and other materials	27	Report which of the following are publicly available and where they can be found: template data collection forms; data extracted from included studies; data used for all analyses; analytic code; any other materials used in the review.	Supplementary file

From: Page MJ, McKenzie JE, Bossuyt PM, Boutron I, Hoffmann TC, Mulrow CD, et al. The PRISMA 2020 statement: an updated guideline for reporting systematic reviews. BMJ 2021;372: n71. doi:10.1136/bmj.n71

Table S3. Search strategy used in each database.

Database	Search strategy
Pubmed-Medline	("strength training"[All Fields] OR "resistance training"[All Fields] OR "weight training"[All Fields]) AND ("hypoxia"[MeSH Terms] OR "hypoxia"[All Fields] OR "hypoxia s"[All Fields] OR "hypoxias"[All Fields] OR ("altitude"[MeSH Terms] OR "altitude"[All Fields] OR "altitudes"[All Fields]) OR (("hypoxia"[MeSH Terms] OR "hypoxia"[All Fields] OR "hypoxic"[All Fields] OR "hypoxical"[All Fields] OR "hypoxically"[All Fields]) AND ("education"[MeSH Subheading] OR "education"[All Fields] OR "training"[All Fields] OR "education"[MeSH Terms] OR "train"[All Fields] OR "train s"[All Fields] OR "trained"[All Fields] OR "training s"[All Fields] OR "trainings"[All Fields] OR "trains"[All Fields])))
Web of Science	(TS=((("strength training") OR ("resistance training") OR ("weight training")))) AND TS=(((hypoxia) OR (altitude) OR (hypoxic training)))
Sport Discuss	(("strength training") OR ("resistance training") OR ("weight training")) AND ((hypoxia) OR (altitude) OR (hypoxic training))
Scopus	TITLE-ABS-KEY ((("strength training") OR ("resistance training") OR ("weight training")) AND ((hypoxia) OR (altitude) OR (hypoxic AND training)))

Table S4. Risk of bias and quality assessment.

Study	Specification of eligibility criteria	Random sequence generation	Allocation concealment	Inter-group similarity at baseline	Blinding of participants	Blinding of outcome data	Incomplete outcome data	Selective reporting	Total quality score
Chycki (2016)	Low	Low	N/A	Low	Low	N/A	Low	High	5/6
Fashi (2020)	Low	Low	N/A	N/A	Low	N/A	Low	Low	5/5
Friedman (2003)	Low	Low	N/A	Low	Low	N/A	Low	High	5/6
Ho (2014)	Low	N/A	N/A	Low	Low	N/A	Low	Low	5/5
Honda (2020)	Low	Low	N/A	Low	N/A	N/A	Low	Low	5/5
Inness (2016)	Low	N/A	N/A	N/A	Low	N/A	Low	High	3/4
Kon (2014)	Low	Low	N/A	Low	N/A	N/A	Low	Low	5/5
Kurobe (2015)	Low	Low	N/A	Low	Low	N/A	Low	Low	6/6
Manimmanakorn (2013)	Low	Low	N/A	N/A	N/A	N/A	Low	Low	4/4
Martínez-Guardado (2019)	Low	Low	N/A	Low	Low	N/A	Low	Low	6/6
Martínez-Guardado (2020)	Low	Low	N/A	N/A	Low	N/A	Low	Low	5/5
Mayo (2018)	Low	Low	N/A	Low	Low	N/A	Low	Low	6/6
Nishimura (2010)	Low	Low	N/A	Low	High	N/A	Low	Low	5/6
Ramos-Campo (2019)	Low	Low	N/A	Low	Low	N/A	Low	Low	6/6
Törpel (2020)	Low	Low	Low	High	Low	Low	Low	High	6/8
van Doorslaer de ten Ryen (2021)	Low	Low	N/A	Low	Low	N/A	Low	Low	6/6
Yan (2016)	Low	Low	N/A	N/A	Low	N/A	Low	Low	5/5

Low: Low risk of bias; High: High risk of bias; Unclear: unclear risk of bias. N/A: not applicable.

Table S5. Equations used for the calculation of effect sizes.

	H group versus N group	Post versus Pre training
**Standardized mean change**	$d = c(df_{H,N}) \cdot \left[\frac{(\bar{X}_{pre,H} - \bar{X}_{pos,H}) - (\bar{X}_{pre,N} - \bar{X}_{pos,N})}{\bar{S}_{pre}}\right]$	$d = c(df) \cdot \left[\frac{\bar{X}_{pre,H} - \bar{X}_{pos,H}}{S_{pre}}\right]$
**Mean baseline standard deviation**	$\bar{S}_{pre} = \sqrt{\frac{(n_H - 1) \cdot S^2{}_{pre,H} + (n_N - 1) \cdot S^2{}_{pre,N}}{n_H + n_N - 2}}$	
**Correction factor**	$c(df_{H,N}) = 1 - \frac{3}{4(n_H + n_N - 2) - 1}$	$c(df) = 1 - \left(\frac{3}{4(n - 1) - 1}\right)$
**Variance[a]**	$S^2(d) = [c(df_{H,N})]^2 \cdot 2(1-r) \cdot \left(\frac{n_H + n_N}{n_H n_N}\right) \cdot \left(\frac{n_H + n_N - 2}{n_H + n_N - 4}\right) \cdot \left[1 + \frac{n_H \cdot n_N \cdot d^2}{2(1-r)(n_H + n_N)}\right] - d^2$	$S^2(d) = [c(df)]2 \cdot \left[\frac{2 \cdot (1-r)}{n}\right] \cdot \left(\frac{n-1}{n-3}\right) \cdot \left[1 + \frac{n \cdot d^2}{2 \cdot (1-r)}\right] - d^2$

N = Normoxic group; H = Hypoxic group; d = standardized effect size; c(df) = correction factor; X = mean; $\bar{S}_{pre}$ = mean baseline standard deviation; n = sample size; S^2 = Variance;
[a] r = 0.7, according to the standard r value recommended by Rosenthal [1].

1. Rosenthal R. Meta-analytic procedures for social research. Newbury Park, CA: Sage 1991.

Figure S1. R code for the statistical analysis and forest plot.

```
library(readxl)
Metanalisis_R <- read_excel("Metanalisis R.xlsx",
                            sheet = "RCTs RM")
library(metafor)
# Statistical analysis for all the measure variables
dat <- Metanalisis_R
yi <- dat$yi
vi <- dat$vi
res <- rma(yi, vi, measure = "GEN", level = 90)
dat$weights <- paste0(round(weights(res)), "%")
mlabfun <- function(text, res) {
  list(bquote(paste(.(text),
                    " Q = ", .(formatC(res$QE, digits=2, format="f")),
                    ", df = ", .(res$k - res$p),
                    ", p ", .(metafor:::.pval(res$QEp, digits=2, showeq=TRUE, sep=" ")), "; ",
                    I^2, " = ", .(formatC(res$I2, digits=1, format="f")), "%, ",
                    tau^2, " = ", .(formatC(res$tau2, digits=2, format="f")))))}
# Use in case of multilevel analysis
# res <- rma.mv(yi, vi, level = 90, random = ~ 1 | studyid/esid, data=dat)
# dat$weights <- paste0(round(weights(res, type = "rowsum"), digits = 1), "%")
# I2 <- (res$sigma2[2])/(res$sigma2[1]+res$sigma2[2]+mean(dat$vi))*100
# mlabfun <- function(text, res) {
              list(bquote(paste(.(text),
                    " Q = ", .(formatC(res$QE, digits=2, format="f")),
                    ", df = ", .(res$k - res$p),
                    ", p ", .(metafor:::.pval(res$QEp, digits=2, showeq=TRUE, sep=" ")), "; ",
                    I^2, " = ", .(formatC(I2, digits=1, format="f")), "% ")))}

#forest plot without subanalysis (RM example)
forest(res, xlim=c(-15, 6.5), ylim=c(-2, 30),
       at=(c(-2, 0, 2.5)),
       header = FALSE,
       cex = 0.95,
```

```
        xlab="Standardized Mean Difference",
        rows= 1:27,
        slab = dat$Author,
ilab=cbind(format(round(dat$aE,1)),format(round(dat$n_E,1)),format(round(dat$aC,1)),format(round(dat$n_C,1)),format(round(dat$aCambio,1)),format(round(dat$Spre,1)),dat$weights),
        ilab.xpos=c(-8.5, -7.5, -6, -5, -3.5, -2.5, 3),
        mlab=mlabfun("RE Model for All Studies", res))
text(c(-8.5, -7.5, -6, -5, -3.5, -2.5, 3), 29, c("ΔH", "n", "ΔN", "n", "ΔH-ΔN", "Spre", "Weight"))
text(c(-8,-5.5), 30, c("H group", "N group"))
text(-15, 29, "Study", pos=4)
text(4.5, 30, "Std. MD", pos=4)
text(6.5, 29, "Random [90%CI]", pos=2)
text(-15, -2, pos=4, cex=0.95, bquote(paste("Test for overall effect: ",
                                Z, " = ", .(formatC(res$zval, digits=2, format="f")),
                                ", p = ", .(formatC(res$pval, digits=2, format="f")))))

#forest plot (subanalysis example; severity of hypoxia in RM)
forest(res, xlim=c(-15, 6.5), ylim=c(-2, 38),
        at=(c(-2, 0, 2.5)),
        order = dat$Severity,
        header = FALSE,
        cex = 0.85,
        xlab="Standardized Mean Difference",
        rows=c(4:12, 17:34),
        slab = dat$Author,
ilab=cbind(format(round(dat$aE,1)),format(round(dat$n_E,1)),format(round(dat$aC,1)),format(round(dat$n_C,1)),format(round(dat$aCambio,1)),format(round(dat$Spre,1)),dat$weights),
        ilab.xpos=c(-8.5, -7.5, -6, -5, -3.5, -2.5, 3),
        mlab=mlabfun("RE Model for All Studies", res))
text(c(-8.5, -7.5, -6, -5, -3.5, -2.5, 3), 37, c("ΔH", "n", "ΔN", "n", "ΔH-ΔN", "Spre", "Weight"))
text(c(-8,-5.5), 38, c("H group", "N group"))
text(-15, 37, "Study", pos=4)
text(4.5, 38, "Std. MD", pos=4)
text(6.5, 37, "Random [90%CI]", pos=2)
text(-15, 35, "Moderate (14.3 - 16 %FiO2)", pos=4, font = 4, cex = 0.95)
```

```
text(-15, 13, "High (<14.3 - 11 %FiO2)", pos=4, font = 4, cex = 0.95)

res.m <- rma(yi, vi, level=90, subset=(Severity=="Mid"), data=dat)

res.h <- rma(yi, vi, level=90, subset=(Severity=="High"), data=dat)

addpoly(res.m, level=90, row=15.5, cex=0.85, efac=1.5, width=c(4,5,4),
mlab=mlabfun("Heterogenity:", res.m))

addpoly(res.h, level=90, row= 2.5, cex=0.85, efac=1.5, width=c(4,5,4),
mlab=mlabfun("Heterogenity:", res.h))

text(-15, 14.5, pos=4, cex=0.85, bquote(paste("Test for overall effect: ",

                                   Z, " = ", .(formatC(res.m$zval, digits=2, format="f")),

                                   ", p = ", .(formatC(res.m$pval, digits=2, format="f")))))

text(-15, 1.5, pos=4, cex=0.85, bquote(paste("Test for overall effect: ",

                                   Z, " = ", .(formatC(res.h$zval, digits=2, format="f")),

                                   ", p = ", .(formatC(res.h$pval, digits=2, format="f")))))

text(-15, -2, pos=4, cex=0.85, bquote(paste("Test for overall effect: ",

                                   Z, " = ", .(formatC(res$zval, digits=2, format="f")),

                                   ", p = ", .(formatC(res$pval, digits=2, format="f")))))

# Use in case of multilevel analysis in subanalysis

#res.m <- rma.mv(yi, vi, level=90, subset=(Severity=="Mid"), random = ~ 1 | studyid/esid,
data=dat)

#res.h <- rma.mv(yi, vi, level=90, subset=(Severity=="High"), random = ~ 1 | studyid/esid,
data=dat)

#I2m <- (res.m$sigma2[2])/(res.m$sigma2[1]+res.m$sigma2[2]+mean(dat$vi,
subset=(Severity=="Mid")))*100

#I2h <- (res.h$sigma2[2])/(res.h$sigma2[1]+res.h$sigma2[2]+mean(dat$vi,
subset=(Severity=="High")))*100

#mlabfunm <- function(text, res) {

  list(bquote(paste(.(text),

                " Q = ", .(formatC(res$QE, digits=2, format="f")),

                ", df = ", .(res$k - res$p),

                ", p ", .(metafor:::.pval(res$QEp, digits=2, showeq=TRUE, sep=" ")), "; ",

                I^2, " = ", .(formatC(I2m, digits=1, format="f")), "% ")))}

#mlabfunh <- function(text, res) {

  list(bquote(paste(.(text),

                " Q = ", .(formatC(res$QE, digits=2, format="f")),

                ", df = ", .(res$k - res$p),

                ", p ", .(metafor:::.pval(res$QEp, digits=2, showeq=TRUE, sep=" ")), "; ",
```

```
I^2, " = ", .(formatC(I2h, digits=1, format="f")), "% ")))}

#addpoly(res.m, level=90, row=15.5, cex=0.85, efac=1.5, width=c(4,5,4),
mlab=mlabfunm("Heterogenity:", res.m))

#addpoly(res.h, level=90, row= 2.5, cex=0.85, efac=1.5, width=c(4,5,4),
mlab=mlabfunh("Heterogenity:", res.h))

#text(-15, 14.5, pos=4, cex=0.85, bquote(paste("Test for overall effect: ",
                    Z, " = ", .(formatC(res.m$zval, digits=2, format="f")),
                    ", p = ", .(formatC(res.m$pval, digits=2, format="f")))))

#text(-15, 1.5, pos=4, cex=0.85, bquote(paste("Test for overall effect: ",
                    Z, " = ", .(formatC(res.h$zval, digits=2, format="f")),
                    ", p = ", .(formatC(res.h$pval, digits=2, format="f")))))

#text(-15, -2, pos=4, cex=0.85, bquote(paste("Test for overall effect: ",
                    Z, " = ", .(formatC(res$zval, digits=2, format="f")),
                    ", p = ", .(formatC(res$pval, digits=2, format="f")))))
```

Figure S2. Funnel plot of the analyzed variables.

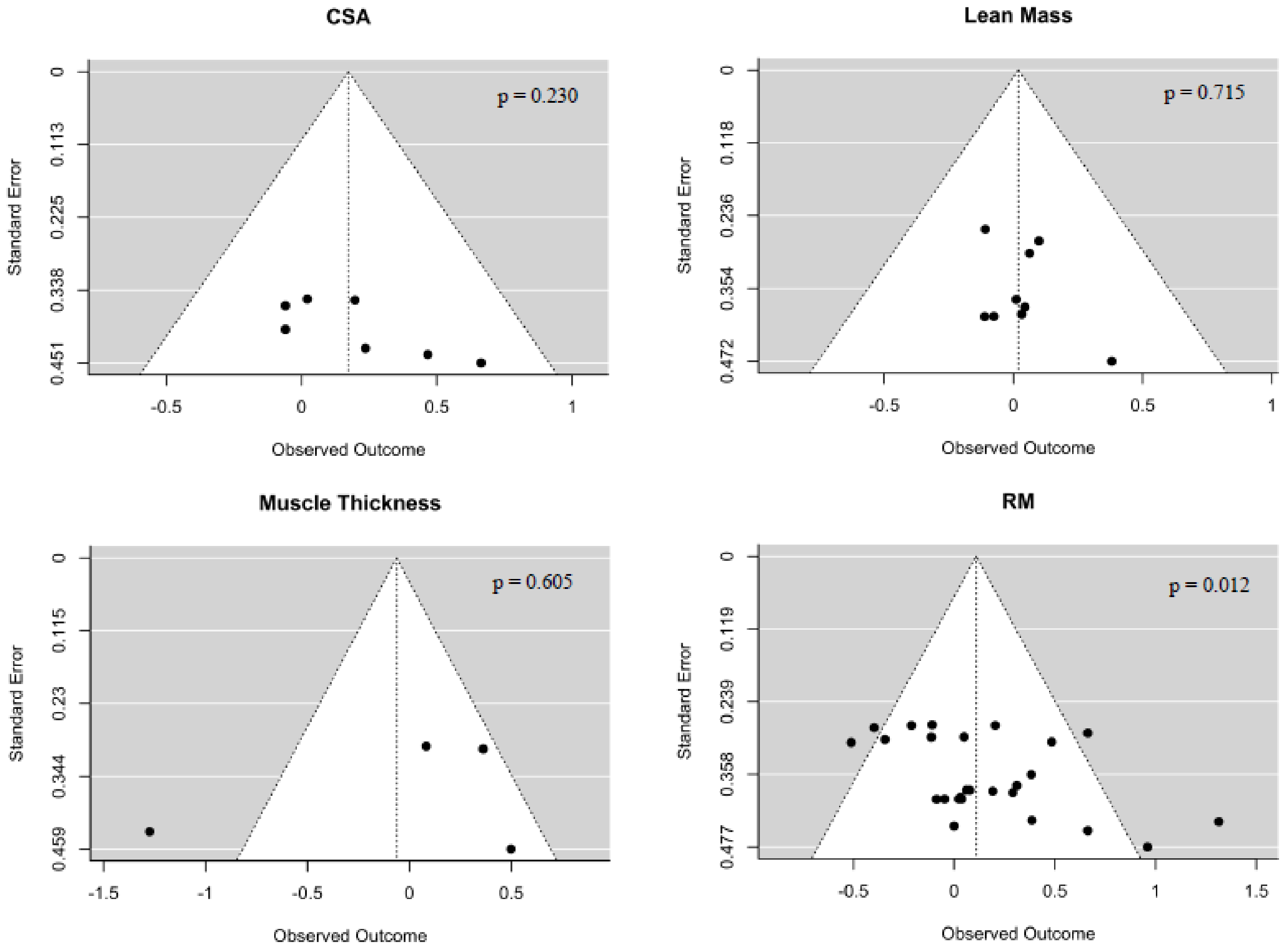

Supplement: Supplementary file 1 — Supplementary Information. [file 41598_2023_30808_MOESM1_ESM.pdf]
